# Association of cortical microstructure with amyloid-β and tau: impact on cognitive decline, neurodegeneration, and clinical progression in older adults

**DOI:** 10.1038/s41380-021-01290-z

**Published:** 2021-09-29

**Authors:** Elena Rodriguez-Vieitez, Victor Montal, Jorge Sepulcre, Cristina Lois, Bernard Hanseeuw, Eduard Vilaplana, Aaron P. Schultz, Michael J. Properzi, Matthew R. Scott, Rebecca Amariglio, Kathryn V. Papp, Gad A. Marshall, Juan Fortea, Keith A. Johnson, Reisa A. Sperling, Patrizia Vannini

**Affiliations:** 1 Massachusetts General Hospital, Harvard Medical School, Boston, MA USA; 2 Athinoula A. Martinos Center for Biomedical Imaging, Charlestown, MA USA; 3 Department of Neurobiology, Care Sciences and Society, Karolinska Institutet, Stockholm, Sweden; 4 Sant Pau Memory Unit, Department of Neurology, Hospital de la Santa Creu i Sant Pau, Biomedical Research Institute Sant Pau, Universitat Autònoma de Barcelona, Barcelona, Spain; 5 Centre of Biomedical Investigation Network for Neurodegenerative Diseases (CIBERNED), Madrid, Spain; 6 Gordon Center for Medical Imaging, Boston, MA USA; 7 Saint Luc University Hospital, Université Catholique de Louvain, Brussels, Belgium; 8 Brigham and Women’s Hospital, Harvard Medical School, Boston, MA USA

**Keywords:** Diagnostic markers, Psychiatric disorders, Prognostic markers, Neuroscience, Psychology

## Abstract

Noninvasive biomarkers of early neuronal injury may help identify cognitively normal individuals at risk of developing Alzheimer’s disease (AD). A recent diffusion-weighted imaging (DWI) method allows assessing cortical microstructure via cortical mean diffusivity (cMD), suggested to be more sensitive than macrostructural neurodegeneration. Here, we aimed to investigate the association of cMD with amyloid-β and tau pathology in older adults, and whether cMD predicts longitudinal cognitive decline, neurodegeneration and clinical progression. The study sample comprised *n* = 196 cognitively normal older adults (mean[SD] 72.5 [9.4] years; 114 women [58.2%]) from the Harvard Aging Brain Study. At baseline, all participants underwent structural MRI, DWI, ^11^C-Pittsburgh compound-B-PET, ^18^F-flortaucipir-PET imaging, and cognitive assessments. Longitudinal measures of Preclinical Alzheimer Cognitive Composite-5 were available for *n* = 186 individuals over 3.72 (1.96)-year follow-up. Prospective clinical follow-up was available for *n* = 163 individuals over 3.2 (1.7) years. Surface-based image analysis assessed vertex-wise relationships between cMD, global amyloid-β, and entorhinal and inferior-temporal tau. Multivariable regression, mixed effects models and Cox proportional hazards regression assessed longitudinal cognition, brain structural changes and clinical progression. Tau, but not amyloid-β, was positively associated with cMD in AD-vulnerable regions. Correcting for baseline demographics and cognition, increased cMD predicted steeper cognitive decline, which remained significant after correcting for amyloid-β, thickness, and entorhinal tau; there was a synergistic interaction between cMD and both amyloid-β and tau on cognitive slope. Regional cMD predicted hippocampal atrophy rate, independently from amyloid-β, tau, and thickness. Elevated cMD predicted progression to mild cognitive impairment. Cortical microstructure is a noninvasive biomarker that independently predicts subsequent cognitive decline, neurodegeneration and clinical progression, suggesting utility in clinical trials.

## Introduction

Alzheimer’s disease (AD) is characterized by the misfolding and deposition of amyloid-β (Aβ) and hyperphosphorylated tau in the brain [1, 2], a process that begins years before clinical onset [3]. Accumulating evidence from preclinical and clinical studies supports the notion that Aβ and tau pathologies interact synergistically in the preclinical stages of AD, contributing to faster neurodegeneration and cognitive decline [4–7]. Therefore, in vivo imaging biomarkers of AD proteinopathy, neuronal injury and neurodegeneration are of interest to elucidate the dynamic interplay among biological mechanisms underlying disease progression.

Although Aβ positivity and even sub-threshold Aβ load have been shown to predict cognitive decline happening over longer time periods [8–10], Aβ alone is not accurate enough to predict short-term cognitive decline or clinical progression [11, 12]. Therefore, complementary noninvasive imaging biomarkers of subtle neuronal injury—whether alone or in combination with Aβ– may help select participants at the earliest stages with an enhanced risk of impending cognitive decline or clinical progression. Biomarkers for identification of at-risk individuals prior to widespread neurodegeneration are of great interest for optimization of secondary prevention trials [13–15], and as outcome measures of therapeutic efficacy.

While neurodegeneration is typically reflected in macrostructural changes including atrophy and cortical thinning measured by structural MRI, a recent diffusion-weighted imaging (DWI) method has allowed to assess microstructural properties of the gray matter (GM) [16, 17] by means of cortical mean diffusivity (cMD). Increased cMD is thought to reflect the early breakdown of microstructural integrity due to damage to cellular membranes and dendritic processes [18], and therefore cMD has been proposed as a sensitive biomarker of subtle microstructural injury, prior to overt neurodegeneration measured by atrophy or cortical thinning [19, 20]. Previous cross-sectional studies have reported increased cMD in prodromal and dementia stages of sporadic AD [16, 21], and a positive association between cMD values and years to symptom onset in autosomal dominant AD [17, 22, 23]. In sporadic fronto-temporal degeneration and amyotrophic lateral sclerosis, increased cMD was more widespread, had a larger effect size and was more closely associated with disease severity compared with cortical thinning [24, 25]. Increased cMD has also been observed in AD-vulnerable regions in pre-dementia stages of AD at pathological levels of CSF Aβ and phospho-tau [16]. However, the relationship between cMD and the underlying in vivo Aβ and tau burden in cognitively normal adults is unknown. Moreover, the ability of cMD to predict subsequent cognitive decline, neurodegeneration, and clinical progression in the AD continuum remains unknown.

The specific aims of this study are to: (i) investigate the cross-sectional association of in vivo Aβ and tau burden with cMD in a cohort of cognitively normal older adults, (ii) determine whether baseline cMD is associated with prospective longitudinal cognitive change and hippocampal atrophy rates, independently and/or interactively with Aβ and tau at baseline, and (iii) determine whether baseline cMD predicts subsequent clinical progression.

## Participants and methods

### Study design and participants

The study sample consisted of *n* = 196 community-dwelling older adults from the Harvard Aging Brain Study (HABS) (Table 1), a longitudinal observational study of aging and preclinical AD conducted at Massachusetts General Hospital and Brigham and Women’s Hospital in Boston, MA [26].

**Table 1:** Characteristics of the study sample.

Characteristic	All participants (*n* = 196)	Aβ− (*n* = 147)	Aβ+ (*n* = 49)	*P* value
No. (% of sample)				
Female, no. (%)	114 (58.2%)	86 (58.5%)	28 (57.1%)	0.87
White/non-hispanic, no. (%)	148 (75.5%)	105 (71.4%)	43 (87.8%)	0.02
* APOE*-ε4+	53 (27.0%)	23 (15.6%)	30 (61.2%)	<0.001
CDR = 0.5	9 (4.6%)	8 (5.4%)	1 (2.0%)	0.32
Mean (SD)				
Age, years	72.5 (9.4)	70.9 (9.6)	77.3 (7.2)	<0.001
Years of education	16.2 (2.9)	16.2 (3.0)	16.0 (2.8)	0.63
MMSE	29.1 (1.1)	29.1 (1.1)	29.2 (1.1)	0.80
Logical memory, delayed recall	15.7 (3.9)	15.7 (3.9)	15.7 (3.9)	>0.99
PACC5	0.19 (0.75)	0.22 (0.75)	0.09 (0.74)	0.30
PIB-FLR DVR	1.17 (0.19)	1.08 (0.04)	1.45 (0.19)	<0.001
entFTP PVC SUVr	1.36 (0.29)	1.29 (0.23)	1.56 (0.34)	<0.001
i-tFTP PVC SUVr	1.44 (0.18)	1.40 (0.15)	1.56 (0.21)	<0.001
No. follow-up MRI scans Prospective MRI follow-up, years Subsample *n*/total *n* (%)	1.47 (0.52) 3.11 (1.52) 118/196 (60.2%)	1.44 (0.50) 3.01 (1.50) 79/147 (53.7%)	1.51 (0.56) 3.31 (1.55) 39/49 (79.6%)	0.49 0.31
No. follow-up cognitive assessments Prospective cognitive follow-up, years Subsample *n*/total *n* (%)	3.56 (1.80) 3.72 (1.96) 186/196 (94.9%)	3.40 (1.81) 3.50 (1.92) 138/147 (93.9%)	4.04 (1.71) 4.34 (1.95) 48/49 (98.0%)	0.03 0.01
Prospective clinical follow-up, years Subsample *n*/total *n* (%) Progressors to MCI, *n*/total (%) Time-to-progression in those who progressed, years	3.2 (1.7) 163/196 (83.2%) 11/163 (6.7%) 3.3 (1.5)	3.1 (1.7) 116/147 (78.9%) 1/116 (0.86%) 4.5 (−)	3.4 (1.6) 47/49 (95.9%) 10/47 (21.3%) 3.2 (1.5)	0.30
Prospective CDR follow-up, years Subsample *n*/total *n* (%) Progressors to CDR = 0.5, *n*/total (%) Time-to-CDR = 0.5 in those who progressed, years	3.3 (1.7) 165/187 (88.2%) 15/165 (9.1%) 2.7 (1.7)	3.3 (1.6) 119/139 (85.6%) 3/119 (2.5%) 3.4 (0.9)	3.3 (1.8) 46/48 (95.8%) 12/46 (26.1%) 2.5 (1.9)	>0.99

Participant information is presented for the full sample and at two levels of Aβ burden. Statistical differences between the Aβ+ and the Aβ− groups were computed using two-sample *t* tests or chi-square *t* tests, as appropriate. *APOE*-ε4 data were missing for 12 participants.

*APOE* apolipoprotein, *CDR* Clinical Dementia Rating, *DVR* distribution volume ratio, *entFTP* entorhinal ^18^F-flortaucipir, *i-tFTP* inferior-temporal ^18^F-flortaucipir, *FTP* ^18^F-flortaucipir, *PIB-FLR* frontal, lateral temporo-parietal and retrosplenial composite PIB-PET, *MCI* mild cognitive impairment, *MMSE* mini-mental state examination, *PACC5* Preclinical Alzheimer Cognitive Composite-5, *PVC* partial volume corrected, *SD* standard deviation, *SUVr* standardized uptake value ratio.

For the aims of this study, we selected participants with concurrent data on DWI, T1-weighted MRI, ^11^C-Pittsburgh Compound-B (PIB)-PET, ^18^F-flortaucipir (FTP)-PET, and cognitive assessments summarized using the Preclinical Alzheimer Cognitive Composite-5 (PACC5) [27]. All assessments had been performed within 1 year of the T1-weighted MRI scan. Using these inclusion criteria, we got a group of *n* = 196 participants (referred to as “baseline”), all deemed cognitively normal. Of note, the majority of participants had a Clinical Dementia Rating (CDR) = 0, except for nine participants with CDR = 0.5. Subsets of the cohort had longitudinal MRI, neuropsychological, CDR and clinical evaluations (Table 1). Ethical approvals, exclusion criteria, and neuropsychological evaluations are detailed in Supplementary Methods.

### MRI methods

All participants underwent a structural 3D T1-weighted magnetization-prepared rapid-acquisition gradient-echo (MPRAGE) sequence and a DWI sequence on a 3-Tesla TimTrio scanner (Siemens, Erlangen, Germany) with a 12-channel phased-array head coil (acquisition parameters in Supplementary Methods).

Structural MRI was processed for estimation of cortical thickness (CTh) and subcortical volumetric segmentation using FreeSurfer 6.0 (http://surfer.nmr.mgh.harvard.edu) [28]. Cortical segmentations were visually inspected to detect and correct processing errors and an automatic region-of-interest (ROI) parcellation was performed [29]. Hippocampal volume (HV), adjusted for intracranial volume, was assessed using Freesurfer.

DWI data were processed with an in-house surface-based diffusion tensor imaging (DTI) approach combining FSL (FMRIB Software Library) (http://fsl.fmrib.ox.ac.uk/fsl/fslwiki, v5.0.9) and FreeSurfer 6.0 tools [16]. This surface-based approach applies recently developed techniques [30–32] to overcome limitations of traditional voxel-based approaches. First, it reduces the contribution from CSF and white matter signal on GM voxels that can confound cMD measures. Second, it applies a surface-based smoothing procedure, less sensitive to smoothing kernel size compared with voxel-based analyses [33]. In the surface-based DTI approach, images were motion-corrected via rigid-body registration between the b = 0 and the 30 b = 700 volumes. After removing non-brain tissue, a tensor model was fitted using FSL’s *dtifit* command, and we computed the cMD metric. The diffusion images were then coregistered to each subject’s T1 using *bbregister*, a boundary-based registration algorithm in FreeSurfer [34]. The cMD maps resulting from DTI fitting were then sampled in the midpoint between white and pial surfaces, projected onto the subject’s cortical surface space, and registered to FreeSurfer standard space. Finally, cMD maps were normalized to a standard surface template (fsaverage) and smoothed using a 15-mm 2D full-width half-maximum Gaussian kernel across the cortical mantle. cMD was extracted from eight AD-vulnerable ROIs: entorhinal, fusiform gyrus, inferior-temporal, middle-temporal, inferior-parietal, orbitofrontal, isthmus cingulate and parahippocampal gyrus. These eight ROIs are typically described as vulnerable to tau aggregation based on postmortem and in vivo staging [2, 35, 36] and data-driven approaches [37].

### PET methods

All PIB and FTP-PET scans were acquired at the Massachusetts General Hospital PET facility (ECAT EXACT HR+ scanner; Siemens, Erlangen, Germany) [26, 38]; (acquisition parameters described in Supplementary Methods). Late-sum PIB and FTP-PET images were used to coregister the respective PET volumes to each subject’s native T1 using *mri_coreg* in FreeSurfer, prior to quantification.

#### PIB-PET quantification

A Logan model was applied to dynamic PIB-PET images using cerebellar GM as reference to generate parametric non-partial volume corrected (non-PVC) Logan distribution volume ratio (DVR) images, which were projected onto the cortical surface. Individual Αβ burden was extracted from a cortical composite including frontal, lateral-temporal, parietal and retrosplenial (PIB-FLR) regions [38, 39]. Participants were further stratified into Aβ+ and Aβ− subgroups at baseline using non-PVC PIB-FLR Logan DVR = 1.2 as cutoff, previously derived using Gaussian mixture modeling [12].

#### Tau PET quantification

FTP-PET images were projected onto the cortical surface space and partial volume corrected using the geometric transfer matrix method [40]. FTP-PET was then quantified using PVC standardized uptake value ratio (SUVr) using cerebellar GM as reference. Individual tau burden was extracted from the bilateral entorhinal and inferior-temporal cortices. Entorhinal FTP (entFTP) was used as a proxy for aging and early tau deposition in preclinical AD, while inferior-temporal FTP (i-tFTP) represents AD-related neocortical tau [5, 38, 41, 42].

### Statistical analyses

#### Cross-sectional analyses

##### Surface-based analyses

To investigate the associations of Aβ and tau with cortical microstructure, we applied a general linear model in FreeSurfer with vertex-wise cMD as dependent variable in three models. For each model, the independent predictor was global Aβ burden evaluated in the PIB-FLR cortical composite, entFTP or i-tFTP; age and sex were covariates. Vertex-wise analyses were corrected for multiple comparisons within FreeSurfer using a cluster extension criterion based on Monte Carlo simulation with 10,000 repeats, with family-wise error correction at *P* < 0.05, two-sided test.

##### Regional-based analyses

Separate multivariable regression models were used to independently assess the ability of global Aβ burden (continuous or dichotomous PIB-FLR), entFTP, and i-tFTP to predict regional cMD. Also, models were set-up to examine the interaction of global Aβ and either entFTP or i-tFTP in predicting regional cMD, and their respective independent contributions. All models included age and sex as covariates. Corrections for multiple regional comparisons were performed using a false discovery rate (*q* < 0.05) approach, two-sided test.

#### Longitudinal analyses

##### Prediction of cognitive decline and neurodegeneration rates

To investigate whether regional cMD is associated with longitudinal cognitive and neurodegeneration rates, the longitudinal changes in PACC5 or HV were extracted from mixed effects models with PACC5 or HV as outcome, using time (years from baseline) as fixed effects predictor, and incorporating random intercepts and slopes at the subject level (Eq. (1)). From these models, we extracted an individual random slope of PACC5 and of HV for each participant, which represented the corresponding rates of change [43]:

1 $$Longitudinal\;PACC5\;or\;HV\,\sim\, time_{from\;baseline} \,+\, (time_{from\;baseline}|{{{{{\rm{subject}}}}}}\;ID)$$

Multivariable regression models were then used to investigate whether regional cMD at baseline predicts slope of PACC5 or HV. All models included age and sex as covariates; in models predicting cognitive decline, baseline PACC5 and education were also added as covariates. Furthermore, we tested whether cMD interacted with dichotomous or continuous PIB-FLR, entFTP, or i-tFTP on the slope of PACC5 or HV; models with interaction terms included all lower-order terms.

We further applied a hierarchical regression approach to test the ability of cMD to predict PACC5 or HV slopes when sequentially including PIB-FLR and CTh as independent predictors, and finally adding entFTP or i-tFTP as predictors. The statistical fit of different models was inter-compared using R^2^ and Akaike information criterion (AIC); lowest AIC indicates better fit. All statistical tests were two-sided.

##### Prediction of clinical outcomes

We used survival analysis to investigate whether cMD predicts subsequent clinical progression. Time-to-event was defined as years from baseline to the first follow-up visit when a participant was diagnosed as mild cognitive impairment (MCI). For comparison, separate survival models were set-up using progression from CDR = 0 to 0.5 as a subtler definition of clinical progression [44]. We applied multivariable Cox proportional hazards regression models to estimate hazard ratios (HRs) with 95% confidence intervals (CI) to investigate whether dichotomous regional cMD (“high cMD” defined as top-tertile cMD; “low cMD” otherwise) predicts subsequent diagnosis of MCI/AD, or progression to CDR = 0.5. Cox regression analyses were controlled for baseline age, sex and education, to account for demographic differences across participants. Additional exploratory Cox regression analyses sequentially incorporated PIB status and CTh as predictors. Results were visualized using Kaplan–Meier curves. All statistical tests were two-sided. Further details about statistical tests and software are included in Supplementary Methods.

## Results

Demographic and clinical data of the 196 participants with baseline data stratified into Aβ+ and Aβ− subgroups are presented in Table 1. Aβ+ participants had greater prevalence of *APOE*-ε4 positivity, were older and had greater Aβ and tau pathology than the Aβ− subgroup; there were no significant differences in cognitive performance between Aβ+ and Aβ− subgroups at baseline.

### Cross-sectional associations of in vivo Aβ and tau with cMD in older adults

No significant association was found between PIB-FLR and vertex-wise cMD after multiple-comparisons correction. In the ROI-based analysis, neither dichotomous nor continuous PIB-FLR were associated with regional cMD, corrected for multiple comparisons.

**Fig. 1:**
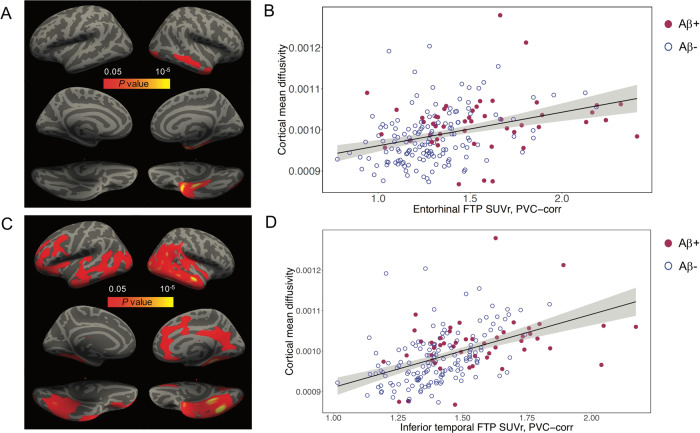
Cross-sectional associations of entorhinal and inferior-temporal tau with cMD. **A** Surface-based statistical map representing the clusters with significant association of vertex-wise cMD with entorhinal ^18^F-flortaucipir (FTP) uptake; clusters survived correction for multiple-comparisons implemented in FreeSurfer by using a cluster extension criterion in a Monte Carlo simulation with 10,000 repeats, with the family-wise error correction settled at *P* < 0.05. **B** Scatterplot illustrating the association of cMD in the middle-temporal gyrus region-of-interest (ROI) with entorhinal FTP uptake. **C** Surface-based statistical map representing the clusters with significant association of vertex-wise cMD with inferior-temporal FTP uptake; clusters survived correction for multiple-comparisons implemented in FreeSurfer by using a cluster extension criterion in a Monte Carlo simulation with 10,000 repeats, with the family-wise error correction settled at *P* < 0.05. **D** Scatterplot illustrating the association of milddle-temporal cMD with inferior-temporal FTP uptake. cMD cortical mean diffusivity, FTP ^18^F-flortaucipir, PVC partial volume corrected, SUVr standardized uptake value ratio.

Entorhinal and inferior-temporal tau (entFTP, i-tFTP) showed a positive cross-sectional association with vertex-wise cMD across all *n* = 196 participants (Fig. 1). The association of entFTP with vertex-wise cMD was localized to clusters in the entorhinal and inferior-middle-temporal gyrus on the right hemisphere (Fig. 1A), while i-tFTP was associated with more widespread increases in cMD in bilateral clusters of entorhinal, isthmus cingulate, fusiform gyrus, inferior-middle-temporal gyrus, and parts of lateral occipital, lateral orbitofrontal cortex and precuneus (Fig. 1C). These associations were confirmed when using ROI-based analyses for eight bilateral cMD ROIs (Supplementary Table 1). Both entFTP and i-tFTP had the strongest positive association with inferior-middle-temporal gyrus cMD, where regression models explained up to 40–45% of the total variance in cMD (Supplementary Table 1). Scatterplots for the ROI-based analyses are illustrated for the middle-temporal gyrus (Fig. 1B, D). There was no significant interaction between entFTP or i-tFTP and either dichotomous or continuous PIB-FLR in predicting concurrent regional cMD (not shown). When either dichotomous or continuous PIB-FLR and entFTP or i-tFTP were entered as independent predictors, the PIB-FLR term was nonsignificant, while the predictive ability of entFTP or i-tFTP was not substantially altered (not shown).

### Relationship between baseline cMD and subsequent rate of cognitive decline

**Fig. 2:**
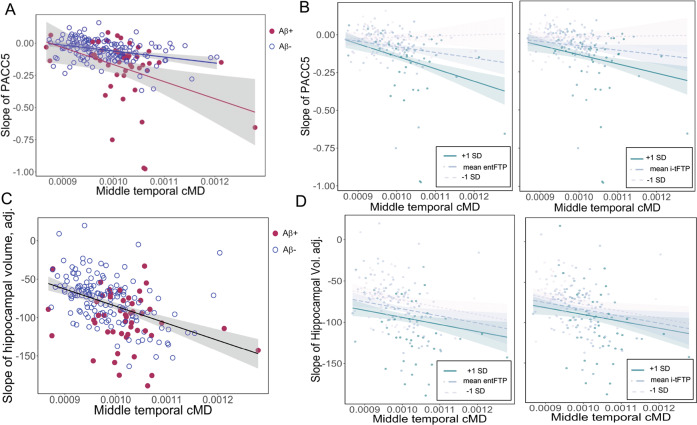
Associations of cMD with subsequent rates of cognitive decline and hippocampal volume loss. **A** Association of middle-temporal cMD at baseline with subsequent rate of cognitive decline as measured by the Preclinical Alzheimer Cognitive Composite-5 (PACC5), illustrating the significant interaction between cMD and dichotomized Aβ burden. **B** Middle-temporal cMD interacts with tau burden (entFTP, i-tFTP) in predicting future rate of cognitive decline. **C** Association of middle-temporal cMD at baseline with subsequent rate of hippocampal volume loss. **D** Middle-temporal cMD does not significantly interact with tau burden (entFTP, i-tFTP) in predicting future rate of hippocampal volume loss. cMD cortical mean diffusivity, entFTP entorhinal ^18^F-flortaucipir, i-tFTP inferior-temporal ^18^F-flortaucipir, PACC5 Preclinical Alzheimer Cognitive Composite-5.

Correcting for baseline demographics and cognitive status (PACC5), baseline cMD in all ROIs predicted steeper decline in PACC5 (Supplementary Table 2); illustrated in the lateral middle-temporal gyrus (Fig. 2A). In five ROIs (Table 2), cMD remained a significant predictor of PACC5 slope after correcting for PIB-FLR, and after simultaneously correcting for both PIB-FLR and regional CTh, indicating that baseline cMD is capturing variance in subsequent cognitive decline, independently from Aβ and CTh biomarkers. The observation that CTh was a nonsignificant predictor in four of the five ROIs (Table 2) supports the concept that cMD has higher sensitivity than CTh as a prognostic marker of cognitive decline. When entFTP was additionally included as predictor, cMD in the isthmus cingulate cortex remained a significant predictor of cognitive decline (Supplementary Table 3); when i-tFTP was used as predictor instead of entFTP, none of the regional cMD values remained predictive of cognitive decline, suggesting that the shared variance between cMD and subsequent cognitive decline is explained by increased neocortical tau pathology.

**Table 2:** Multivariable regression models predicting the rate of cognitive decline.

Indep. pred.	Std. β (95% CI)	*P* value	*q* value	*R*^2^ (AIC)	Indep. pred.	Std. β (95% CI)	*P* value	*q* value	*R*^2^ (AIC)
	**Slope PACC5 ~ fusiform cMD** **+** **PIB-FLR** **+** **PACC5** **+** **(fusiform CTh)**								
Fusiform cMD	−0.24 (−0.38 to −0.10)	8 × 10^−4^	0.005	0.34 (489)	Fusiform cMD	−0.17 (−0.32 to −0.03)	0.022	0.043	0.37 (484)
PIB-FLR	−0.33 (−0.45 to −0.21)	2 × 10^−7^	3 × 10^−7^		PIB-FLR	−0.32 (−0.44 to −0.20)	3 × 10^−7^	4 × 10^−7^	
PACC5	0.27 (0.14 to 0.40)	9 × 10^−5^	1 × 10^−4^		PACC5	0.25 (0.12 to 0.38)	2 × 10^−4^	3 × 10^−4^	
					Fusiform CTh	0.17 (0.04 to 0.30)	0.012	0.046	
	**Slope PACC5 ~ inferior-temporal cMD** **+** **PIB-FLR** **+** **PACC5** **+** **(inferior-temporal CTh)**								
Inf. temp. cMD	−0.23 (−0.39 to −0.08)	0.003	0.005	0.34 (491)	Inf. temp. cMD	−0.20 (−0.37 to −0.03)	0.020	0.043	0.34 (492)
PIB-FLR	−0.34 (−0.47 to −0.22)	8 × 10^−8^	2 × 10^−7^		PIB-FLR	−0.35 (−0.47 to −0.23)	6 × 10^−8^	2 × 10^−7^	
PACC5	0.27 (0.14 to 0.41)	6 × 10^−5^	1 × 10^−4^		PACC5	0.27 (0.14 to 0.40)	8 × 10^−5^	2 × 10^−4^	
					Inf. temp. CTh	0.06 (−0.08 to 0.19)	0.40	0.48	
	**Slope PACC5 ~ isthmus cingulate cMD** **+** **PIB-FLR ** **+** ** PACC5 ** **+** **(isthmus cingulate CTh)**								
Isthmus cing. cMD	−0.21 (−0.34 to −0.08)	0.001	0.005	0.34 (490)	Isthmus cing. cMD	−0.21 (−0.34 to −0.08)	0.001	0.011	0.34 (491)
PIB-FLR	−0.34 (−0.46 to −0.22)	1 × 10^−7^	2 × 10^−7^		PIB-FLR	−0.34 (−0.46 to −0.21)	2 × 10^−7^	3 × 10^−7^	
PACC5	0.26 (0.13 to 0.39)	1 × 10^−4^	1 × 10^−4^		PACC5	0.26 (0.13 to 0.39)	2 × 10^−4^	2 × 10^−4^	
					Isthmus cing. CTh	0.04 (−0.09 to 0.16)	0.57	0.57	
	**Slope PACC5 ~ lateral orbitofrontal cMD** **+** ** PIB-FLR** **+** ** PACC5** **+** **(lateral orbitofrontal CTh)**								
Lateral orbitofr. cMD	−0.15 (−0.28 to −0.01)	0.034	0.039	0.32 (496)	Lateral orbitofr. cMD	−0.16 (−0.29 to −0.02)	0.027	0.044	0.32 (497)
PIB-FLR	−0.35 (−0.47 to −0.23)	6 × 10^−8^	2 × 10^−7^		PIB-FLR	−0.35 (−0.48 to −0.23)	6 × 10^−8^	2 × 10^−7^	
PACC5	0.28 (0.14 to 0.41)	6 × 10^−5^	1 × 10^−4^		PACC5	0.28 (0.15 to 0.42)	5 × 10^−5^	2 × 10^−4^	
					Lateral orbitofr. CTh	−0.05 (−0.17 to 0.07)	0.42	0.48	
	**Slope PACC5 ~ middle-temporal cMD ** **+ ** **PIB-FLR** **+ ** **PACC5** **+** **(middle-temporal CTh)**								
Mid. temp. cMD	−0.24 (−0.39 to −0.09)	0.002	0.005	0.34 (490)	Mid. temp. cMD	−0.21 (−0.37 to −0.04)	0.016	0.043	0.34 (492)
PIB-FLR	−0.34 (−0.46 to −0.22)	1 × 10^−7^	2 × 10^−7^		PIB-FLR	−0.34 (−0.46 to −0.22)	1 × 10^−7^	2 × 10^−7^	
PACC5	0.26 (0.13 to 0.40)	1 × 10^−4^	1 × 10^−4^		PACC5	0.26 (0.13 to 0.39)	1 × 10^−4^	2 × 10^−4^	
					Mid. temp. CTh	0.06 (−0.08 to 0.21)	0.37	0.48	

All models were adjusted for age, sex and education, which were all nonsignificant predictors. Multiple-comparisons corrected results are indicated by FDR *q* values.

*AIC* Akaike Information Criterion, *cMD* cortical mean diffusivity, *CTh* cortical thickness, *PACC5* Preclinical Alzheimer Cognitive Composite-5, *PIB-FLR* ^11^C-Pittsburgh compound-B in a cortical composite including frontal, lateral temporo-parietal, and retrosplenial regions.

### Cortical mean diffusivity is synergistic with amyloid-β and tau burden in predicting future cognitive decline

We observed a significant interaction between cMD and continuous or dichotomous PIB-FLR in predicting PACC5 slope, which was significant in all ROIs after multiple-comparisons correction (Supplementary Table 4). This interaction is illustrated in Fig. 2A where, as middle-temporal cMD increases, PACC5 declines with a steeper slope in the Aβ+ compared with the Aβ− subgroup. The interaction between cMD and continuous PIB-FLR is illustrated in Supplementary Fig. 1, which represents the association of baseline middle-temporal cMD with PACC5 slope for mean PIB-FLR ± 1 standard deviation (SD) range. In these interaction models, the individual terms for PIB-FLR and regional cMD (in all ROIs except entorhinal and inferior-parietal) remained significant independent predictors (Supplementary Table 4).

There was also a significant interaction between regional cMD and tau burden as measured by either entFTP or i-tFTP in predicting the rate of cognitive decline, as illustrated in Fig. 2B for cMD in the middle-temporal region. In these interaction models, the individual terms for entFTP, i-tFTP, and regional cMD (in fusiform, isthmus cingulate and parahippocampus) remained significant independent predictors (Supplementary Table 5).

### Relationship between baseline cMD and subsequent rate of HV loss

Regional cMD in all ROIs predicted the rate of HV loss, after multiple-comparisons correction (Supplementary Table 6), illustrated in Fig. 2C for the middle-temporal gyrus cMD. The ability of regional cMD to predict rate of HV loss remained significant after correcting for PIB-FLR, regional CTh, and either entFTP (Supplementary Table 7) or i-tFTP (Supplementary Table 8).

Regional cMD did not interact with PIB-FLR (not shown). Also, the interaction of cMD with continuous measures of tau burden (entFTP or i-tFTP) was nonsignificant in predicting the rate of HV loss (Fig. 2D).

### Cortical mean diffusivity is predictive of subsequent clinical progression

**Fig. 3:**
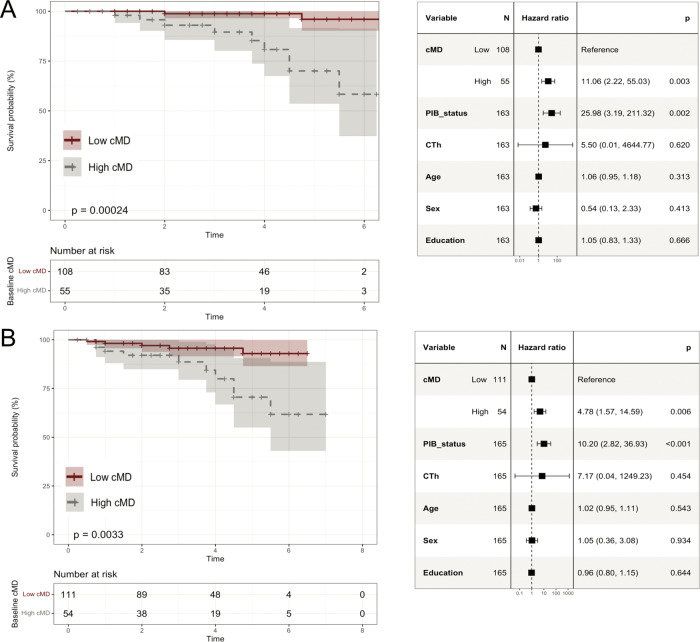
Survival analyses illustrating the ability of cMD to predict subsequent clinical progression. Kaplan–Meier curves and Cox proportional hazards regression results for lateral orbitofrontal cMD (high/low groups) predicting (**A**) progression to MCI, (**B**) progression to CDR = 0.5. cMD cortical mean diffusivity, CTh cortical thickness, PIB_status Aβ+ vs. Aβ−; “High cMD” = top-tertile cMD values (“Low cMD”, otherwise).

Eleven participants (6.7% [11/163]) progressed to a clinical diagnosis of MCI within a mean (SD) progression time of 3.3 (1.5) years. Using CDR as outcome, we found that 15 participants (9.1% [15/165]) progressed to CDR = 0.5 during 2.7 (1.7) years. Despite few participants progressed clinically during the study period, we observed that higher cMD predicted faster progression. In particular, entorhinal, middle-temporal and orbitofrontal cMD predicted shorter survival using MCI or CDR = 0.5 as outcome, as illustrated in Fig. 3 for the orbitofrontal cMD. Cox proportional hazard models included cMD, PIB status and CTh as independent predictors; all models were covaried by age, sex and education (Fig. 3). In these clinical progression models, PIB status was found to be the strongest predictor of clinical progression, which showed an HR [95% CI] = 25.98 [3.19 to 211.32], *P* = 0.002 in predicting progression to MCI and HR [95% CI] = 10.20 [2.82 to 36.93], *P* < 0.001 in predicting progression to CDR = 0.5. Orbitofrontal cMD remained significant after inclusion of PIB status and CTh as independent predictors (Fig. 3). Orbitofrontal cMD showed an HR [95% CI] = 11.06 [2.22 to 55.03], *P* = 0.003 in predicting progression to MCI, and HR [95% CI] = 4.78 [1.57 to 14.59], *P* = 0.006 in predicting progression to CDR = 0.5. CTh did not significantly predict clinical progression in any of the models (Fig. 3). Respective survival analyses in entorhinal and middle-temporal gyrus are illustrated in Supplementary Figs. 2, 3.

## Discussion

In this study of 196 older adults, entorhinal and inferior-temporal tau, but not global Aβ, were positively associated with cMD in AD-vulnerable brain areas. Increased cMD at baseline predicted faster cognitive decline, which remained significant after correction for global Aβ, regional CTh, and entorhinal tau. We also observed a synergistic interaction between cMD and global Aβ, and between cMD and tau burden, on subsequent rate of cognitive decline. Higher cMD at baseline predicted faster hippocampal atrophy and clinical progression to MCI. At baseline, entorhinal and inferior-temporal tau were positively associated with regional cMD, suggesting that elevated cMD is a marker of neuronal injury accompanying tau as it spreads into the neocortex. Our finding that increased cMD is associated with tau but not Aβ burden is consistent with the independent roles that these proteinopathies play in the brain [4, 45]. Recent studies [16, 17, 46] reported a non-linear effect of Aβ load on structural biomarkers in the brain in clinically-normal individuals prior to overt neuronal damage, which could explain the lack of a linear association between cMD and global Aβ in our cohort.

We then assessed the relationship between baseline cMD and subsequent cognitive decline, neurodegeneration and clinical progression. We found that higher cMD values were strongly predictive of steeper cognitive decline and HV loss. The ability of regional cMD to predict subsequent cognitive decline remained significant after sequentially accounting for global Aβ burden, regional CTh and entorhinal tau, suggesting that cMD independently explains variance in cognitive decline, beyond those traditional imaging biomarkers. Moreover, regional cMD did not significantly predict subsequent cognitive decline once inferior-temporal tau was included as independent predictor. Our findings suggest that the shared variance between cMD and subsequent cognitive decline may be explained by increasing neocortical tau pathology, which is likely an underlying biological substrate driving the elevated cMD signal. Our results are in line with accumulating evidence that, while entorhinal tau can increase with age, inferior-temporal tau is a stronger predictor of subsequent AD-specific cognitive decline [47, 48]. In previous cross-sectional studies in patients with sporadic AD, fronto-temporal degeneration and amyotrophic lateral sclerosis [19, 20, 24, 25], cMD was associated with cognitive performance independently from CTh. Our study extends our knowledge about cMD to cognitively unimpaired individuals, where we found that cMD has prognostic ability to predict short-term cognitive decline beyond that provided by Aβ, structural biomarkers and entorhinal tau.

The regions where increased cMD was predictive of cognitive decline independently from CTh are consistent with regions undergoing hypometabolism in preclinical AD, in particular the isthmus cingulate located next to the posterior cingulate cortex [41], where a synergistic contribution of Aβ and tau leads to metabolic dysfunction in the absence of atrophy. Together with those previous reports, our findings support the notion that both cMD and ^18^F-fluorodeoxyglucose-PET are early biomarkers of tau-driven synaptic dysfunction preceding overt neuronal death and atrophy. Increased cMD in the absence of cortical thinning might also reflect early microstructural damage in response to tau oligomers that cause synaptic toxicity and dysfunction prior to neuronal death as demonstrated in preclinical studies [4], or to the accumulation of other protein oligomers or deposits of TDP-43 or α-synuclein, for which no PET tracers are yet available.

Our study also showed that cMD and either Aβ or tau burden contribute independently and synergistically to subsequent cognitive decline over ~3.5 years. This finding suggests that in a clinical trial selecting Aβ+ participants [13, 15], the addition of the cMD biomarker would help to further select those participants most likely to decline over a relatively short term. Our finding adds to current efforts to compare the utility of different biomarkers of Aβ, tau and neurodegeneration, whether used alone or in combination, to predict short-term cognitive decline and clinical progression [44, 49]. In line with previous observations [8], we found that PIB status was a strong predictor of clinical progression to MCI or CDR = 0.5 during ~3.5 years follow-up. In addition, a novel finding of our study was that higher cMD independently predicted faster clinical progression to MCI or CDR = 0.5, beyond that predicted by PIB status, while CTh did not contribute with any significant predictive value. These findings add support to the concept that cMD has higher sensitivity than CTh in the AD continuum and that it may be a useful biomarker for stratification of at-risk individuals for prevention trials that typically extend over 3 to 5 years.

Regional cMD showed prognostic ability for subsequently faster rate of hippocampal atrophy, demonstrating added value beyond imaging biomarkers of global Aβ, CTh, and entorhinal or inferior-temporal tau; the absence of a synergistic effect between cMD and either Aβ or tau might be explained because longitudinal MRI data was available for only ~60% of baseline participants, having fewer longitudinal follow-ups compared to the more comprehensive longitudinal cognitive and clinical data available. Alternatively, the presence of co-pathologies might in part explain the observed hippocampal atrophy. Previous reports have suggested that, while hippocampal atrophy is a rather specific feature of underlying AD pathology [50], neurodegeneration in this brain region may be due to a confluence of multiple underlying AD and non-AD co-pathologies including TDP-43 [51]. In particular, postmortem TDP-43 burden was found to be associated with antemortem hippocampal atrophy as measured with MRI, independently from Aβ and tau pathology [52]. Based on these previous reports, we acknowledge that the cMD signal in our study may be partly due to underlying TDP-43 pathology that contributes to HV loss independently from Aβ and tau pathology, and which may be associated with a slower rate of cognitive decline than AD pathology.

While our study focused on microstructural properties in the GM, three previous studies investigated white matter microstructural changes in the same cohort [53–55]. Increased mean diffusivity in the hippocampal cingulum white matter bundle was associated with greater downstream tau pathology in the posterior cingulate cortex, an effect that was enhanced at high levels of Aβ burden, and suggesting that white matter tracts might serve as pathways for tau propagation [53]. Our results are in line with Jacobs et al. [53] with one key difference in that we studied microstructural properties in the GM. Our study found that AD pathology, in particular tau, is associated with microstructural injury in the GM. Further longitudinal studies are needed to investigate whether changes in cMD in the GM are locally and/or distally associated to longitudinal accumulation of tau, and whether they temporally precede or follow from degeneration of white matter tracts. A related study in older adults from HABS [54] found that global fractional anisotropy (FA) averaged across nine white matter tracts predicted longitudinal cognitive decline independently but not synergistically with Aβ burden. A follow-up study [55] reported that the synergistic interaction between FA in the fornix and Aβ burden was associated with subsequently faster episodic memory decline. Similar to these reports [54, 55], we found that cMD predicted subsequent rate of cognitive decline independently and synergistically with Aβ status. From our results we conclude that in the continuum of AD, and especially in pre-symptomatic Aβ+ individuals, cMD may be a promising marker to identify individuals at enhanced risk of short-term cognitive decline and clinical progression, with utility for prevention trials.

HABS is a cohort study enrolling community-dwelling older adults that are followed over time. Our finding that cMD has prognostic value in a convenience sample such as HABS, not enriched for AD-risk factors, suggests that cMD is a sensitive marker of early and subtle neuronal injury, with promising potential as a prognostic biomarker with higher sensitivity compared with macrostructural biomarkers. Additional studies on the ability of cMD to predict cognitive decline and clinical progression in cohorts enriched for AD-risk factors such as *APOE*-ε4, autosomal dominant mutations, or other more stringent inclusion criteria, would be valuable to confirm our results.

A major strength of our study is its highly multimodal nature including concurrent T1-weighted MRI, DWI, Aβ, and tau neuroimaging data. Also, the cohort was comprehensively characterized in terms of cognitive assessments and multiple longitudinal neuroimaging, neuropsychological, and clinical data points. Since GM is considered mostly isotropic with respect to the motion of water molecules [18, 20], we selected cMD as an optimum metric to assess cortical microstructure in the GM. Although it could be technically possible to compute FA in the GM, its anisotropic-based computation would make it less informative and straightforward to interpret compared with cMD. Our study has some limitations. Only ~60% of the study participants had longitudinal MRI scans, which possibly limited the statistical power of the analyses involving HV loss. We only had longitudinal DWI in a small subset of participants so DWI analyses were restricted to cross-sectional data; further longitudinal investigations would be valuable to explore relationships between changes in cMD, Aβ, and tau to investigate the temporal dynamics of microstructural damage and proteinopathy accumulation.

Our study showed that cortical microstructure is a promising noninvasive technique, sensitive to early microstructural injury in older adults. Given that neuronal loss is irreversible, the ability of cMD to detect subtle microstructural damage prior to overt atrophy may have important clinical implications. We found that tau is an underlying pathological substrate associated with increases in cMD. As such, cMD might be a proxy for tau-induced neuronal injury, and cortical microstructure could serve as a lower-cost, noninvasive alternative to tau PET in clinical settings. The combination of multimodal baseline and longitudinal data allowed us to demonstrate the ability of cMD to predict cognitive and clinical progression using three different independent measures: slope of PACC5 and progression to MCI or to CDR = 0.5; the confirmation of the prognostic ability of cMD using three different methods adds robustness to our findings. The ability of cMD to predict short-term cognitive decline and clinical progression suggests utility as outcome measure and to improve risk stratification of participants in clinical trials.

## Supplementary information

Supplementary information

## References

[1] Arriagada PV, Growdon JH, Hedley-Whyte ET, Hyman BT (1992). Neurofibrillary tangles but not senile plaques parallel duration and severity of Alzheimer’s disease. Neurology..

[2] Braak H, Braak E (1991). Neuropathological stageing of Alzheimer-related changes. Acta Neuropathol.

[3] Jack CR, Bennett DA, Blennow K, Carrillo MC, Dunn B, Haeberlein SB (2018). NIA-AA research framework: toward a biological definition of Alzheimer’s disease. Alzheimers Dement.

[4] Busche MA, Hyman BT (2020). Synergy between amyloid-β and tau in Alzheimer’s disease. Nat Neurosci..

[5] Sperling RA, Mormino EC, Schultz AP, Betensky RA, Papp KV, Amariglio RE (2019). The impact of Aβ and tau on prospective cognitive decline in older individuals. Ann Neurol..

[6] Ossenkoppele R, Smith R, Ohlsson T, Strandberg O, Mattsson N, Insel PS (2019). Associations between tau, Aβ, and cortical thickness with cognition in Alzheimer disease. Neurology..

[7] La Joie R, Visani AV, Baker SL, Brown JA, Bourakova V, Cha J (2020). Prospective longitudinal atrophy in Alzheimer’s disease correlates with the intensity and topography of baseline tau-PET. Sci Transl Med..

[8] Farrell ME, Jiang S, Schultz AP, Properzi MJ, Price JC, Becker JA (2021). Defining the lowest threshold for amyloid-PET to predict future cognitive decline and amyloid accumulation. Neurology..

[9] Donohue MC, Sperling RA, Petersen R, Sun C-K, Weiner MW, Aisen PS (2017). Association between elevated brain amyloid and subsequent cognitive decline among cognitively normal persons. JAMA..

[10] Bischof GN, Jacobs HIL (2019). Subthreshold amyloid and its biological and clinical meaning: long way ahead. Neurology..

[11] Mormino EC, Betensky RA, Hedden T, Schultz AP, Ward A, Huijbers W (2014). Amyloid and APOE ε4 interact to influence short-term decline in preclinical Alzheimer disease. Neurology..

[12] Mormino EC, Betensky RA, Hedden T, Schultz AP, Amariglio RE, Rentz DM (2014). Synergistic effect of β-amyloid and neurodegeneration on cognitive decline in clinically normal individuals. JAMA Neurol..

[13] Sperling RA, Rentz DM, Johnson KA, Karlawish J, Donohue M, Salmon DP (2014). The A4 study: stopping AD before symptoms begin?. Sci Transl Med..

[14] Sperling RA, Aisen PS, Beckett LA, Bennett DA, Craft S, Fagan AM (2011). Toward defining the preclinical stages of Alzheimer’s disease: recommendations from the National Institute on Aging-Alzheimer’s Association workgroups on diagnostic guidelines for Alzheimer’s disease. Alzheimers Dement.

[15] Sperling R, Mormino E, Johnson K (2014). The evolution of preclinical Alzheimer’s disease: implications for prevention trials. Neuron..

[16] Montal V, Vilaplana E, Alcolea D, Pegueroles J, Pasternak O, González-Ortiz S (2018). Cortical microstructural changes along the Alzheimer’s disease continuum. Alzheimers Dement.

[17] Montal V, Vilaplana E, Pegueroles J, Bejanin A, Alcolea D, Carmona-Iragui M (2021). Biphasic cortical macro- and microstructural changes in autosomal dominant Alzheimer’s disease. Alzheimers Dement.

[18] Le Bihan D (2003). Looking into the functional architecture of the brain with diffusion MRI. Nat Rev Neurosci..

[19] Vogt NM, Hunt JF, Adluru N, Dean DC, Johnson SC, Asthana S (2020). Cortical microstructural alterations in mild cognitive impairment and Alzheimer’s disease dementia. Cereb Cortex..

[20] Weston PSJ, Simpson IJA, Ryan NS, Ourselin S, Fox NC (2015). Diffusion imaging changes in grey matter in Alzheimer’s disease: a potential marker of early neurodegeneration. Alzheimers Res Ther.

[21] Torso M, Bozzali M, Zamboni G, Jenkinson M, Chance SA (2021). for the Alzheimer’s disease neuroimage initiative. detection of Alzheimer’s disease using cortical diffusion tensor imaging. Hum Brain Mapp..

[22] Vilaplana E, Rodriguez-Vieitez E, Ferreira D, Montal V, Almkvist O, Wall A (2020). Cortical microstructural correlates of astrocytosis in autosomal-dominant Alzheimer disease. Neurology..

[23] Weston PSJ, Poole T, Nicholas JM, Toussaint N, Simpson IJA, Modat M (2020). Measuring cortical mean diffusivity to assess early microstructural cortical change in presymptomatic familial Alzheimer’s disease. Alzheimers Res Ther..

[24] Illán-Gala I, Montal V, Borrego-Écija S, Vilaplana E, Pegueroles J, Alcolea D (2019). Cortical microstructure in the behavioural variant of frontotemporal dementia: looking beyond atrophy. Brain..

[25] Illán-Gala I, Montal V, Pegueroles J, Vilaplana E, Alcolea D, Dols-Icardo O (2020). Cortical microstructure in the amyotrophic lateral sclerosis-frontotemporal dementia continuum. Neurology..

[26] Dagley A, LaPoint M, Huijbers W, Hedden T, McLaren DG, Chatwal JP (2017). Harvard aging brain study: dataset and accessibility. Neuroimage..

[27] Papp KV, Rentz DM, Orlovsky I, Sperling RA, Mormino EC (2017). Optimizing the preclinical Alzheimer’s cognitive composite with semantic processing: The PACC5. Alzheimers Dement..

[28] Fischl B, Dale AM (2000). Measuring the thickness of the human cerebral cortex from magnetic resonance images. Proc Natl Acad Sci USA.

[29] Desikan RS, Ségonne F, Fischl B, Quinn BT, Dickerson BC, Blacker D (2006). An automated labeling system for subdividing the human cerebral cortex on MRI scans into gyral based regions of interest. Neuroimage..

[30] Beer AL, Plank T, Meyer G, Greenlee MW (2013). Combined diffusion-weighted and functional magnetic resonance imaging reveals a temporal-occipital network involved in auditory-visual object processing. Front Integr Neurosci..

[31] Wu M, Lu LH, Lowes A, Yang S, Passarotti AM, Zhou XJ (2014). Development of superficial white matter and its structural interplay with cortical gray matter in children and adolescents. Hum Brain Mapp..

[32] Coalson TS, Van Essen DC, Glasser MF (2018). The impact of traditional neuroimaging methods on the spatial localization of cortical areas. Proc Natl Acad Sci USA..

[33] Jones D, Symms M, Cercignani M, Howard R (2005). The effect of filter size on VBM analyses of DT-MRI data. Neuroimage..

[34] Greve DN, Fischl B (2009). Accurate and robust brain image alignment using boundary-based registration. Neuroimage..

[35] Jack CR, Wiste HJ, Weigand SD, Therneau TM, Lowe VJ, Knopman DS (2017). Defining imaging biomarker cut points for brain aging and Alzheimer’s disease. Alzheimers Dement.

[36] Schöll M, Lockhart SN, Schonhaut DR, O’Neil JP, Janabi M, Ossenkoppele R (2016). PET imaging of tau deposition in the aging human brain. Neuron..

[37] Sepulcre J, Grothe MJ, Sabuncu M, Chhatwal J, Schultz AP, Hanseeuw B (2017). Hierarchical organization of tau and amyloid deposits in the cerebral cortex. JAMA Neurol.

[38] Johnson KA, Schultz A, Betensky RA, Becker JA, Sepulcre J, Rentz D (2016). Tau positron emission tomographic imaging in aging and early Alzheimer disease. Ann Neurol.

[39] Johnson KA, Gregas M, Becker JA, Kinnecom C, Salat DH, Moran EK (2007). Imaging of amyloid burden and distribution in cerebral amyloid angiopathy. Ann Neurol.

[40] Rousset OG, Ma Y, Evans AC (1998). Correction for partial volume effects in PET: principle and validation. J Nucl Med..

[41] Hanseeuw BJ, Betensky RA, Schultz AP, Papp KV, Mormino EC, Sepulcre J (2017). Fluorodeoxyglucose metabolism associated with tau-amyloid interaction predicts memory decline. Ann Neurol..

[42] Scott MR, Hampton OL, Buckley RF, Chhatwal JP, Hanseeuw BJ, Jacobs HIL (2020). Inferior temporal tau is associated with accelerated prospective cortical thinning in clinically normal older adults. Neuroimage..

[43] Hanseeuw BJ, Betensky RA, Jacobs HIL, Schultz AP, Sepulcre J, Becker JA (2019). Association of amyloid and tau with cognition in preclinical Alzheimer disease: a longitudinal study. JAMA Neurol.

[44] Rabin JS, Neal TE, Nierle HE, Sikkes SAM, Buckley RF, Amariglio RE (2020). Multiple markers contribute to risk of progression from normal to mild cognitive impairment. NeuroImage Clin.

[45] van der Kant R, Goldstein LSB, Ossenkoppele R (2020). Amyloid-β-independent regulators of tau pathology in Alzheimer disease. Nat Rev Neurosci..

[46] Harrison TM, Du R, Klencklen G, Baker SL, Jagust WJ (2021). Distinct effects of beta‐amyloid and tau on cortical thickness in cognitively healthy older adults. Alzheimers Dement.

[47] Cho H, Choi JY, Lee HS, Lee JH, Ryu YH, Lee MS (2019). Progressive tau accumulation in Alzheimer disease: 2-year follow-up study. J Nucl Med..

[48] Ossenkoppele R, Smith R, Mattsson-Carlgren N, Groot C, Leuzy A, Strandberg O (2021). Accuracy of tau positron emission tomography as a prognostic marker in preclinical and prodromal Alzheimer disease: a head-to-head comparison against amyloid positron emission tomography and magnetic resonance imaging. JAMA Neurol..

[49] Papp KV, Buckley R, Mormino E, Maruff P, Villemagne VL, Masters CL (2020). Clinical meaningfulness of subtle cognitive decline on longitudinal testing in preclinical AD. Alzheimers Dement.

[50] Jack CR, Dickson DW, Parisi JE, Xu YC, Cha RH, O’Brien PC (2002). Antemortem MRI findings correlate with hippocampal neuropathology in typical aging and dementia. Neurology..

[51] Wilson RS, Yu L, Trojanowski JQ, Chen E-Y, Boyle PA, Bennett DA (2013). TDP-43 pathology, cognitive decline, and dementia in old age. JAMA Neurol..

[52] Bejanin A, Murray ME, Martin P, Botha H, Tosakulwong N, Schwarz CG (2019). Antemortem volume loss mirrors TDP-43 staging in older adults with non-frontotemporal lobar degeneration. Brain..

[53] Jacobs HIL, Hedden T, Schultz AP, Sepulcre J, Perea RD, Amariglio RE (2018). Structural tract alterations predict downstream tau accumulation in amyloid-positive older individuals. Nat Neurosci..

[54] Rabin JS, Perea RD, Buckley RF, Neal TE, Buckner RL, Johnson KA (2019). Global white matter diffusion characteristics predict longitudinal cognitive change independently of amyloid status in clinically normal older adults. Cereb Cortex..

[55] Rabin JS, Perea RD, Buckley RF, Johnson KA, Sperling RA, Hedden T (2019). Synergism between fornix microstructure and beta amyloid accelerates memory decline in clinically normal older adults. Neurobiol Aging.

