# Supplementary material for: Association of cortical microstructure with amyloid-β and tau: impact on cognitive decline, neurodegeneration, and clinical progression in older adults
Source: Mol Psychiatry. 2021 Sep 29;26(12):7813–22. doi: 10.1038/s41380-021-01290-z (PMC8873001; doi:10.1038/s41380-021-01290-z)
Supplement: Supplementary file 1 [file 41380_2021_1290_MOESM1_ESM.pdf]

Supplementary information

Association of cortical microstructure with amyloid-β and tau: impact on cognitive decline, neurodegeneration and clinical progression in older adults

Rodriguez-Vieitez E†, Montal V†, Sepulcre J, Lois C, Hanseeuw B, Vilaplana E, Schultz AP, Properzi MJ, Scott MR, Amariglio R, Papp KV, Marshall GA, Fortea J, Johnson KA, Sperling RA, Vannini P

†These authors contributed equally to this work.

Supplementary methods

Supplementary tables

Supplementary Table 1. Cross-sectional association of entorhinal and inferior temporal ^{18}F-flortaucipir PET with regional cMD.

Supplementary Table 2. Associations of regional baseline cMD with longitudinal cognitive decline as measured by slope of PACC5.

Supplementary Table 3. Associations of isthmus cingulate cMD with longitudinal cognitive decline as measured by slope of PACC5.

Supplementary Table 4. Interactive effects of regional cMD and global amyloid-β on the rate of cognitive decline.

Supplementary Table 5. Interactive effects of regional cMD and tau burden on the rate of cognitive decline.

Supplementary Table 6. Associations of regional cMD at baseline with longitudinal decline in hippocampal volume.

Supplementary Table 7. Associations of regional cMD at baseline with longitudinal decline in hippocampal volume, accounting for entorhinal ^{18}F-flortaucipir.

Supplementary Table 8. Associations of regional cMD at baseline with longitudinal decline in hippocampal volume, accounting for inferior temporal ^{18}F-flortaucipir.

Supplementary figures

Supplementary Fig. 1. Interaction plot illustrating the association of cMD with the rate of cognitive decline at different levels of amyloid-β burden.

Supplementary Fig. 2. Survival analyses illustrating the ability of cMD in entorhinal cortex to predict subsequent clinical progression.

Supplementary Fig. 3. Survival analyses illustrating the ability of cMD in middle temporal cortex to predict subsequent clinical progression.

Supplementary methods

Study design and participants

Ethical approvals: All participants in this prospective cohort study of community-dwelling older adults provided written informed consent prior to undergoing any study procedures. The Harvard Aging Brain Study (HABS) protocol is approved annually by the Partners Human Research Committee.

Characteristics of participants, exclusion criteria and clinical evaluations: This prospective cohort study included longitudinal data collected from 2010-2020. At study entry, participants were excluded if they had a history of alcoholism, drug abuse, or head trauma. All participants were determined to be cognitively normal if they had no current clinical depression (Geriatric Depression Scale [GDS] ≤ 10) or active psychiatric illness, mini-mental state examination (MMSE) ≥ 25, and performance within 1.5 standard deviations (SD) of age and education-adjusted norms on the Wechsler Logical Memory II delayed recall [LM]. In the HABS, the Clinical Dementia Rating (CDR) is completed by neuropsychologists and psychiatrists and rated independently from other cognitive test results, and all CDR raters are blinded to participant biomarker status. At study entry and all subsequent annual follow-up investigations, clinical diagnosis was evaluated in consensus meetings involving six or more experienced clinicians as part of a multidisciplinary team. A participant is discussed if his/her annual CDR score is 0.5 or above and/or performance falls 1.5 SD below the sample mean on any individual domain-specific composite score. A diagnosis of mild cognitive impairment (MCI) or Alzheimer's disease (AD) dementia was determined by clinical consensus after reviewing all available longitudinal CDR, clinical and neuropsychological assessments, and relevant medications/medical history, and blinded to biomarker data, as previously described [1, 2].

To add robustness to our findings regarding prediction of clinical progression, we opted to use two separate methods of assessing clinical progression, as in a previous paper by Papp *et al.* [2], by: 1) using clinical status from the consensus meetings (i.e. from cognitively normal to MCI); 2) the CDR progression (i.e. from CDR = 0 to CDR = 0.5). For Analysis 1), we included the n=9 individuals with CDR = 0.5 at baseline, as long as they had been clinically classified as cognitively normal during the diagnostic consensus meeting. For Analysis 2) CDR is completed by neuropsychologists and psychiatrists and rated independently from other neuropsychological testing results, and blinded to biomarker status of participants [2]. For this analysis, we excluded those participants who had CDR = 0.5 at baseline. In Method 2) we considered that a participant progressed to CDR = 0.5 only when there were at least two CDR = 0.5 measures recorded. This is because the measurement of CDR = 0.5 is an assessment that has some inherent uncertainty, and sometimes participants revert back to CDR = 0.

As ^{18}F-Flortaucipir (FTP) PET was introduced mid-study in HABS, a subset of HABS participants received FTP-PET after their study entry. Self-reported data on race/ethnicity and sex of participants were available as part of the HABS study design.

Available longitudinal data and clinical outcomes: For the 'baseline' cohort in our study (*n*=196), 118 individuals had longitudinal T1-weigthed MRI (at least one follow-up MRI, with an average [SD] of 1.47 [0.52] follow-up MRI scans during 3.11 [1.52] years), and 186 individuals had longitudinal neuropsychological assessments (average [SD] of 3.56 [1.80] visits and 3.72 [1.96] years follow-up) (Table 1).

Annual consensus meetings evaluated progression of participants to MCI or AD dementia (data available in 163 individuals over 3.2 [1.7] years follow-up), and 11/163 (6.7%) participants converted to MCI during the time frame of the study, with an average (SD) time to conversion of 3.3 (1.5) years (Table 1).

As a subtler measure of clinical progression, we recorded when participants progressed from CDR=0 at baseline to a CDR=0.5 at follow-up. After excluding the nine participants with CDR=0.5 at baseline, we had a subset of 165 participants (all with CDR=0 at baseline) who had prospective annual CDR evaluations over an average (SD) time of 3.3 (1.7) years. Of these, 15/165 (9.1%) participants progressed to CDR=0.5 during the longitudinal investigation, with an average time to conversion of 2.7 (1.7) years (Table 1). Because a CDR increase from 0 to 0.5 has been considered as a less stable measure of clinical progression compared to a diagnosis of MCI, we only classified participants as progressors if a CDR=0.5 was obtained at least any two follow-up visits, as previously done [3]. Using this criterion, participants were classified as non-progressors if CDR=0 was obtained at all follow-up visits or if CDR=0.5 was obtained at only one follow-up visit, as previously performed [3]; otherwise, participants were considered to be progressors.

Data availability

Data from HABS are available online at http://nmr.mgh.harvard.edu/lab/harvardagingbrain/data. Longitudinal data will be made available at periodic data releases at the same web address. Qualified investigators must abide by the HABS online data use agreement, designed to protect the privacy of research participants.

Neuropsychological evaluations

Participants in HABS are evaluated annually with a comprehensive battery of neuropsychological assessments including tests of episodic memory, executive function, global cognition and the CDR. For this study, global cognitive performance was assessed with the Preclinical Alzheimer Cognitive Composite-5 (PACC5), a summary measure calculated as a mean of z-score performance on five tests sensitive to cognitive decline in at-risk individuals including global cognition, episodic and semantic memory and executive function domains, as previously described [4], in particular including MMSE (0-30), WMS-R Logical Memory Delayed Recall (LMDR; 0-25), the Digit-Symbol Coding Test (DSC; 0-93), the Free and Cued Selective Reminding Test-Free 1 Total Recall (FCSRT96; 0-96), and category fluency (CAT). The PACC5 is currently used as outcome in both pharmacological and nonpharmacological secondary prevention trials [2].

MRI acquisition parameters

The T1-weighted MPRAGE structural images were collected using the following scan parameters: repetition time (TR) = 2200 ms, echo times (TE) = 1.54, or 3.36, or 5.18, or 7 ms, flip angle=7°, 4× acceleration, 1.0×1.0×1.2 mm voxels. For the diffusion-weighted imaging (DWI) acquisition, a single shot spin echo planar imaging sequence was used with the following parameters: TR=6230, TE=84ms, a flip angle of 90°, FOV: 256×256×128 mm^3; acquired isotropic voxel size 2 mm^3, 30 isotropically distributed diffusion-sensitizing gradients with a b-value of 700 s/mm^2 and five non-diffusion weighted images (b = 0 s/mm^2).

PET acquisition parameters

PIB PET acquisition: Following a transmission scan, 10-15 mCi ^{11}C-PIB was injected intravenously as a bolus and followed immediately by a 60-min dynamic PET scan in 3-D mode (63 image planes, 15.2 cm axial field of view, 5.6 mm transaxial resolution and 2.4 mm slice interval; 69 frames: 12×15s, 57×60s).

Tau PET acquisition: Following a mean (SD) bolus injection of 10.0 (1.0), FTP data were acquired with a 3D list mode, dynamic protocol using the PET camera described above. Images were acquired from 80-100 minutes in 4×5 min frames. All PET data were reconstructed, attenuation corrected and evaluated for head motion.

Statistical tests and software

Statistical analyses were conducted using R statistical software version 3.6.0 (packages: nlme, interactions, survival, ggsurvfit, forestmodel, car, ggplot2; R Foundation for Statistical Computing). Group analyses to compare demographic, neuropsychological, clinical and imaging characteristics between the Aβ+ and Aβ- sub-groups at baseline were performed using parametric t-tests and χ^2 tests for continuous and dichotomous variables, respectively. Statistical significance was given by $P < 0.05$; two-sided tests were used throughout. In ROI-based analyses, statistical results were corrected for multiple comparisons using a false discovery rate (FDR) approach, with statistical significance given by $q < 0.05$. No collinearity was detected in linear regression analyses, as verified using the variance inflation index (VIF); Cox proportional hazards models met the proportionality of hazards assumption.

Supplementary references

1. Dagley A, LaPoint M, Huijbers W, Hedden T, McLaren DG, Chatwal JP, et al. Harvard Aging Brain Study: Dataset and accessibility. Neuroimage. 2017;144:255–258.
2. Papp KV, Buckley R, Mormino E, Maruff P, Villemagne VL, Masters CL, et al. Clinical meaningfulness of subtle cognitive decline on longitudinal testing in preclinical AD. Alzheimers Dement. 2020;16:552–560.
3. Rabin JS, Neal TE, Nierle HE, Sikkes SAM, Buckley RF, Amariglio RE, et al. Multiple markers contribute to risk of progression from normal to mild cognitive impairment. Neuroimage Clin. 2020;28:102400.
4. Papp KV, Rentz DM, Orlovsky I, Sperling RA, Mormino EC. Optimizing the preclinical Alzheimer's cognitive composite with semantic processing: The PACC5. Alzheimers Dement (NY). 2017;3:668–677.

Supplementary Table 1. Cross-sectional association of entorhinal and inferior temporal ^{18}F-flortaucipir PET with regional cMD.

Dependent variable	Indep. pred.	Std. β (95% CI)	*P* value	*q* value	R^2	Indep. pred.	Std. β (95% CI)	*P* value	*q* value	R^2
	**Regional cMD ~ entFTP + age + sex**					**Regional cMD ~ i-tFTP + age + sex**				
Entorhinal cMD	entFTP	0.20 (0.05 to 0.34)	0.009	0.045	0.10	i-tFTP	0.17 (0.02 to 0.33)	0.026	9×10^{-5}	0.09
	Age	0.16 (0.01 to 0.31)	0.037	0.037		Age	0.15 (0.00 to 0.31)	0.051	0.051	
Fusiform cMD	entFTP	0.16 (0.03 to 0.29)	0.014	0.045	0.33	i-tFTP	0.29 (0.17 to 0.42)	1×10^{-5}	9×10^{-5}	0.37
	Age	0.47 (0.34 to 0.60)	1×10^{-11}	3×10^{-11}		Age	0.40 (0.27 to 0.53)	6×10^{-9}	1×10^{-8}	
Inferior parietal cMD	entFTP	0.08 (-0.05 to 0.20)	0.23	0.26	0.35	i-tFTP	0.17 (0.04 to 0.30)	0.010	0.016	0.37
	Age	0.54 (0.41 to 0.66)	8×10^{-15}	2×10^{-14}		Age	0.49 (0.36 to 0.62)	2×10^{-12}	6×10^{-12}	
Inferior temporal cMD	entFTP	0.13 (0.01 to 0.25)	0.030	0.049	0.42	i-tFTP	0.25 (0.13 to 0.37)	5×10^{-5}	2×10^{-4}	0.45
	Age	0.57 (0.45 to 0.69)	2×10^{-17}	2×10^{-16}		Age	0.50 (0.38 to 0.62)	2×10^{-14}	2×10^{-13}	
Isthmus cingulate cMD	entFTP	0.05 (-0.09 to 0.19)	0.52	0.52	0.18	i-tFTP	0.16 (0.02 to 0.31)	0.028	0.032	0.20
	Age	0.39 (0.25 to 0.53)	2×10^{-7}	2×10^{-7}		Age	0.33 (0.19 to 0.48)	1×10^{-5}	1×10^{-5}	
Lateral orbitofront. cMD	entFTP	0.16 (0.03 to 0.29)	0.017	0.045	0.27	i-tFTP	0.15 (0.01 to 0.29)	0.032	0.032	0.27
	Age	0.41 (0.27 to 0.54)	1×10^{-8}	2×10^{-8}		Age	0.40 (0.26 to 0.54)	5×10^{-8}	8×10^{-8}	
Middle temporal cMD	entFTP	0.13 (0.01 to 0.25)	0.030	0.049	0.40	i-tFTP	0.25 (0.13 to 0.37)	8×10^{-5}	2×10^{-4}	0.43
	Age	0.55 (0.43 to 0.67)	2×10^{-16}	1×10^{-15}		Age	0.49 (0.37 to 0.61)	2×10^{-13}	7×10^{-13}	
Parahipp. cMD	entFTP	0.11 (-0.03 to 0.25)	0.12	0.16	0.19	i-tFTP	0.19 (0.05 to 0.33)	0.010	0.016	0.21
	Age	0.39 (0.25 to 0.53)	2×10^{-7}	2×10^{-7}		Age	0.34 (0.20 to 0.49)	5×10^{-6}	7×10^{-6}	

All models were adjusted for sex, which was a non-significant predictor. Multiple-comparisons corrected results are indicated by FDR *q* values.

cMD = cortical mean diffusivity; entFTP = entorhinal ^{18}F-flortaucipir; i-tFTP = inferior temporal ^{18}F-flortaucipir.

Supplementary Table 2. Associations of regional baseline cMD with longitudinal cognitive decline as measured by slope of PACC5.

Independent predictors	Std. β (95% CI)	*P* value	*q* value	R^2 (AIC)
	**Slope PACC5 ~ entorhinal cMD + additional predictors**			
Entorhinal cMD	-0.16 (-0.29 to -0.03)	0.019	0.025	0.21 (523)
PACC5	0.27 (0.13 to 0.42)	2×10^{-4}	3×10^{-4}	
	**Slope PACC5 ~ fusiform cMD + additional predictors**			
Fusiform cMD	-0.29 (-0.44 to -0.14)	2×10^{-4}	0.001	0.24 (514)
PACC5	0.27 (0.13 to 0.41)	2×10^{-4}	3×10^{-4}	
	**Slope PACC5 ~ inf. parietal cMD + additional predictors**			
Inf. parietal cMD	-0.18 (-0.34 to -0.03)	0.024	0.027	0.21 (524)
PACC5	0.28 (0.14 to 0.42)	2×10^{-4}	3×10^{-4}	
	**Slope PACC5 ~ inf. temporal cMD + additional predictors**			
Inf. temporal cMD	-0.26 (-0.42 to -0.10)	0.002	0.003	0.23 (519)
PACC5	0.28 (0.14 to 0.42)	2×10^{-4}	3×10^{-4}	
	**Slope PACC5 ~ isthmus cingulate cMD + additional predictors**			
Isthmus cingulate cMD	-0.24 (-0.38 to -0.10)	7×10^{-4}	0.003	0.23 (517)
PACC5	0.27 (0.13 to 0.41)	2×10^{-4}	3×10^{-4}	
	**Slope PACC5 ~ lateral orbitofrontal cMD + additional predictors**			
Lateral orbitofrontal cMD	-0.16 (-0.31 to -0.01)	0.033	0.033	0.21 (524)
PACC5	0.28 (0.14 to 0.43)	2×10^{-4}	3×10^{-4}	
	**Slope PACC5 ~ middle temporal cMD + additional predictors**			
Middle temporal cMD	-0.27 (-0.43 to -0.11)	0.001	0.003	0.23 (518)
PACC5	0.27 (0.13 to 0.41)	3×10^{-4}	3×10^{-4}	
	**Slope PACC5 ~ parahippocampal cMD + additional predictors**			
Parahippocampal cMD	-0.23 (-0.37 to -0.09)	0.001	0.003	0.23 (518)
PACC5	0.27 (0.12 to 0.41)	3×10^{-4}	3×10^{-4}	

All models were adjusted by age, sex and education, which were non-significant predictors. Multiple-comparisons corrected results are indicated by FDR *q* values.

cMD = cortical mean diffusivity; PACC5 = Preclinical Alzheimer Cognitive Composite-5.

Supplementary Table 3. Associations of isthmus cingulate cMD with longitudinal cognitive decline as measured by slope of PACC5.

Independent predictors	Std. β (95% CI)	*P* value	*q* value	R^2 (AIC)
	**Slope PACC5 ~ isthmus cingulate cMD + additional predictors**			
Isthmus cingulate cMD	-0.21 (-0.33 to -0.09)	6×10^{-4}	0.005	0.45 (459)
PIB-FLR	-0.20 (-0.32 to -0.07)	0.002	0.002	
PACC5	0.23 (0.10 to 0.35)	4×10^{-4}	4×10^{-4}	
Isthmus cingulate CTh	0.04 (-0.08 to 0.15)	0.55	0.63	
entFTP	-0.38 (-0.51 to -0.26)	1×10^{-8}	9×10^{-8}	

Multivariable regression model includes as independent predictors: PIB-FLR, PACC5, isthmus cingulate CTh and entFTP; all models were adjusted by age, sex and education, which were all non-significant predictors. Multiple-comparisons corrected results are indicated by FDR *q* values.

cMD = cortical mean diffusivity; CTh = cortical thickness; entFTP = entorhinal ^{18}F-flortaucipir

Supplementary Table 4. Interactive effects of regional cMD and global amyloid-β on the rate of cognitive decline.

**Slope PACC5 ~ Regional cMD × PIB-FLR + regional cMD + PIB-FLR + PACC5 + age + sex + education**				**Slope PACC5 ~ Regional cMD × PIB (pos/neg) + regional cMD + PIB (pos/neg) + PACC5 + age + sex + education**			
**Interaction and individual terms**	**Std. β (95% CI)**	***P* value**	***q* value**	**Interaction and individual terms**	**Std. β (95% CI)**	***P* value**	***q* value**
Entorhinal				Entorhinal			
cMD × PIB-FLR	-0.20<br>(-0.32 to -0.09)	0.001	0.002	cMD × PIB (pos/neg)	-0.23<br>(-0.34 to -0.12)	2×10^{-4}	5×10^{-4}
cMD	-0.10<br>(-0.22 to 0.02)	0.11	0.11	cMD	-0.10<br>(-0.23 to 0.02)	0.092	0.092
PIB-FLR	-0.28<br>(-0.41 to -0.15)	2×10^{-5}	4×10^{-5}	PIB (pos/neg)	-0.28<br>(-0.41 to -0.16)	1×10^{-5}	8×10^{-5}
Fusiform				Fusiform			
cMD × PIB-FLR	-0.21<br>(-0.32 to -0.10)	8×10^{-4}	0.002	cMD × PIB (pos/neg)	-0.23<br>(-0.35 to -0.11)	2×10^{-4}	6×10^{-4}
cMD	-0.21<br>(-0.35 to -0.08)	0.003	0.010	cMD	-0.23<br>(-0.36 to -0.09)	0.001	0.005
PIB-FLR	-0.26<br>(-0.38 to -0.13)	7×10^{-5}	9×10^{-5}	PIB (pos/neg)	-0.24<br>(-0.36 to -0.11)	3×10^{4}	3×10^{-4}
Inferior parietal				Inferior parietal			
cMD × PIB-FLR	-0.17<br>(-0.28 to -0.06)	0.008	0.008	cMD × PIB (pos/neg)	-0.11<br>(-0.23 to 0.00)	0.07	0.07
cMD	-0.13<br>(-0.27 to 0.02)	0.094	0.11	cMD	-0.13<br>(-0.28 to 0.02)	0.086	0.092
PIB-FLR	-0.30<br>(-0.42 to -0.17)	8×10^{-6}	4×10^{-5}	PIB (pos/neg)	-0.30<br>(-0.42 to -0.17)	1×10^{-5}	8×10^{-5}
Inferior temporal				Inferior temporal			
cMD × PIB-FLR	-0.21<br>(-0.32 to -0.10)	8×10^{-4}	0.002	cMD × PIB (pos/neg)	-0.18<br>(-0.30 to -0.05)	0.006	0.009
cMD	-0.19<br>(-0.34 to -0.04)	0.012	0.020	cMD	-0.19<br>(-0.34 to -0.04)	0.016	0.026
PIB-FLR	-0.28<br>(-0.40 to -0.16)	2×10^{-5}	4×10^{-5}	PIB (pos/neg)	-0.26<br>(-0.39 to -0.13)	1×10^{-4}	2×10^{-4}
Isthmus cing.				Isthmus cing.			
cMD × PIB-FLR	-0.25<br>(-0.38 to -0.12)	6×10^{-5}	5×10^{-4}	cMD × PIB (pos/neg)	-0.21<br>(-0.34 to -0.09)	6×10^{-4}	0.001
cMD	-0.25<br>(-0.38 to -0.13)	$1x10^{-4}$	0.001	cMD	-0.24<br>(-0.37 to -0.11)	4×10^{-4}	0.003
PIB-FLR	-0.26<br>(-0.38 to -0.13)	6×10^{-5}	8×10^{-5}	PIB (pos/neg)	-0.26<br>(-0.39 to -0.14)	4×10^{-5}	2×10^{-4}
Orbitofrontal				Orbitofrontal			
cMD × PIB-FLR	-0.20<br>(-0.33 to -0.06)	0.002	0.003	cMD × PIB (pos/neg)	-0.14<br>(-0.27 to -0.01)	0.029	0.033
cMD	-0.15<br>(-0.29 to -0.02)	0.025	0.034	cMD	-0.14<br>(-0.28 to 0.00)	0.047	0.063
PIB-FLR	-0.29<br>(-0.42 to -0.17)	9×10^{-6}	4×10^{-5}	PIB (pos/neg)	-0.29<br>(-0.42 to -0.16)	2×10^{-5}	9×10^{-5}
Middle temporal				Middle temporal			
cMD × PIB-FLR	-0.21<br>(-0.32 to -0.09)	0.001	0.002	cMD × PIB (pos/neg)	-0.16<br>(-0.28 to -0.05)	0.009	0.012
cMD	-0.21<br>(-0.36 to -0.06)	0.006	0.012	cMD	-0.21<br>(-0.36 to -0.06)	0.007	0.013
PIB-FLR	-0.27<br>(-0.39 to -0.14)	6×10^{-5}	8×10^{-5}	PIB (pos/neg)	-0.27<br>(-0.40 to -0.14)	4×10^{-5}	2×10^{-4}
Parahippocampal				Parahippocampal			
cMD × PIB-FLR	-0.20<br>(-0.32 to -0.07)	0.002	0.003	cMD × PIB (pos/neg)	-0.23<br>(-0.35 to -0.11)	2×10^{-4}	2×10^{-4}
cMD	-0.19<br>(-0.32 to -0.06)	0.005	0.012	cMD	-0.20<br>(-0.33 to -0.08)	0.002	0.005
PIB-FLR	-0.24<br>(-0.38 to -0.11)	4×10^{-4}	4×10^{-4}	PIB (pos/neg)	-0.26<br>(-0.38 to -0.13)	7×10^{-5}	2×10^{-4}

The table shows the statistical results for the 2-way interaction term of regional cMD with continuous PIB-FLR (left) and dichotomous PIB (right), including the effect of each of the independent predictors individually. Multiple-comparisons corrected results are indicated by FDR *q* values.

Supplementary Table 5. Interactive effects of regional cMD and tau burden on the rate of cognitive decline.

**Slope PACC5 ~ Regional cMD × entFTP + regional cMD + entFTP + PACC5 + age + sex + education**				**Slope PACC5 ~ Regional cMD × i-tFTP + regional cMD + i-tFTP + PACC5 + age + sex + education**			
**Interaction and individual terms**	**Std. β (95% CI)**	***P* value**	***q* value**	**Interaction and individual terms**	**Std. β (95% CI)**	***P* value**	***q* value**
Entorhinal				Entorhinal			
cMD × entFTP	-0.21 (-0.32 to -0.10)	4×10^{-4}	9×10^{-4}	cMD × i-tFTP	-0.32 (-0.42 to -0.21)	5×10^{-8}	4×10^{-7}
cMD	-0.06 (-0.17 to 0.06)	0.34	0.34	cMD	-0.10 (-0.21 to 0.01)	0.085	0.17
entFTP	-0.40 (-0.53 to -0.28)	2×10^{-9}	3×10^{-9}	i-tFTP	-0.37 (-0.50 to -0.25)	1×10^{-8}	4×10^{-8}
Fusiform				Fusiform			
cMD × entFTP	-0.22 (-0.34 to -0.11)	1×10^{-4}	5×10^{-4}	cMD × i-tFTP	-0.22 (-0.32 to -0.11)	4×10^{-4}	0.001
cMD	-0.20 (-0.33 to -0.07)	0.003	0.007	cMD	-0.17 (-0.31 to -0.03)	0.020	0.054
entFTP	-0.38 (-0.50 to -0.26)	4×10^{-9}	5×10^{-9}	i-tFTP	-0.34 (-0.47 to -0.20)	2×10^{-6}	2×10^{-6}
Inferior parietal				Inferior parietal			
cMD × entFTP	-0.15 (-0.28 to -0.02)	0.013	0.015	cMD × i-tFTP	-0.09 (-0.21 to 0.02)	0.13	0.13
cMD	-0.12 (-0.26 to 0.01)	0.077	0.10	cMD	-0.08 (-0.23 to 0.07)	0.30	0.30
entFTP	-0.43 (-0.55 to -0.30)	2×10^{-10}	6×10^{-10}	i-tFTP	-0.42 (-0.55 to -0.29)	4×10^{-9}	1×10^{-8}
Inferior temporal				Inferior temporal			
cMD × entFTP	-0.21 (-0.33 to -0.08)	4×10^{-4}	9×10^{-4}	cMD × i-tFTP	-0.20 (-0.32 to -0.08)	0.001	0.002
cMD	-0.14 (-0.28 to 0.00)	0.059	0.094	cMD	-0.10 (-0.26 to 0.05)	0.19	0.22
entFTP	-0.39 (-0.52 to -0.27)	2×10^{-9}	3×10^{-9}	i-tFTP	-0.37 (-0.50 to -0.23)	4×10^{-7}	5×10^{-7}
Isthmus cingulate				Isthmus cing.			
cMD × entFTP	-0.17 (-0.29 to -0.05)	0.004	0.005	cMD × i-tFTP	-0.14 (-0.25 to -0.02)	0.026	0.035
cMD	-0.26 (-0.38 to -0.14)	4×10^{-5}	3×10^{-4}	cMD	-0.20 (-0.33 to -0.07)	0.003	0.013
entFTP	-0.42 (-0.54 to -0.30)	8×10^{-11}	6×10^{-10}	i-tFTP	-0.39 (-0.52 to -0.26)	3×10^{-8}	4×10^{-8}
Orbitofrontal				Orbitofrontal			
cMD × entFTP	-0.08 (-0.20 to 0.04)	0.19	0.19	cMD × i-tFTP	-0.11 (-0.24 to 0.01)	0.059	0.067
cMD	-0.08 (-0.21 to 0.05)	0.24	0.27	cMD	-0.10 (-0.23 to 0.04)	0.16	0.22
entFTP	-0.43 (-0.56 to -0.30)	6×10^{-10}	1×10^{-9}	i-tFTP	-0.43 (-0.56 to -0.29)	2×10^{-9}	1×10^{-8}
Middle temporal				Middle temporal			
cMD × entFTP	-0.19 (-0.31 to -0.07)	0.001	0.002	cMD × i-tFTP	-0.16 (-0.27 to -0.05)	0.013	0.021
cMD	-0.16 (-0.31 to -0.02)	0.025	0.051	cMD	-0.13 (-0.28 to 0.03)	0.11	0.17
entFTP	-0.39 (-0.51 to -0.26)	6×10^{-9}	6×10^{-9}	i-tFTP	-0.37 (-0.50 to -0.23)	5×10^{-7}	6×10^{-7}
Parahippocampal				Parahippocamp.			
cMD × entFTP	-0.22 (-0.34 to -0.10)	8×10^{-5}	5×10^{-4}	cMD × i-tFTP	-0.29 (-0.39 to -0.18)	7×10^{-7}	3×10^{-6}
cMD	-0.19 (-0.31 to -0.07)	0.002	0.007	cMD	-0.18 (-0.31 to -0.06)	0.003	0.013
entFTP	-0.40 (-0.52 to -0.29)	3×10^{-10}	8×10^{-10}	i-tFTP	-0.37 (-0.50 to -0.25)	2×10^{-8}	4×10^{-8}

The table shows the statistical results for the 2-way interaction term of regional cMD with entorhinal ^{18}F-flortaucipir (entFTP, left), and with inferior temporal ^{18}F-flortaucipir (i-tFTP, right), including the effect of each of the independent predictors individually. Multiple-comparisons corrected results are indicated by FDR *q* values.

cMD = cortical mean diffusivity; entFTP = entorhinal ^{18}F-flortaucipir; i-tFTP = inferior temporal ^{18}F-flortaucipir; PACC5 = Preclinical Alzheimer Cognitive Composite-5

Supplementary Table 6. Associations of regional cMD at baseline with longitudinal decline in hippocampal volume.

Independent predictors	Std. β (95% CI)	*P* value	*q* value	R^2 (AIC)
	**Slope HV ~ entorhinal cMD + age + sex**			
Entorhinal cMD	-0.19 (-0.32 to -0.07)	0.003	0.009	0.27 (502)
Age	-0.41 (-0.53 to -0.28)	2×10^{-9}	2×10^{-8}	
	**Slope HV ~ fusiform cMD + age + sex**			
Fusiform cMD	-0.27 (-0.42 to -0.13)	3×10^{-4}	0.002	0.28 (497)
Age	-0.31 (-0.45 to -0.16)	4×10^{-5}	5×10^{-5}	
	**Slope HV ~ inf. parietal cMD + age + sex**			
Inf. parietal cMD	-0.22 (-0.37 to -0.07)	0.005	0.009	0.26 (503)
Age	-0.33 (-0.48 to -0.18)	3×10^{-5}	4×10^{-5}	
	**Slope HV ~ inf. temporal cMD + age + sex**			
Inf. temporal cMD	-0.24 (-0.40 to -0.09)	0.003	0.009	0.27 (501)
Age	-0.30 (-0.46 to -0.15)	2×10^{-4}	2×10^{-4}	
	**Slope HV ~ isthmus cingulate cMD + age + sex**			
Isthmus cingulate cMD	-0.18 (-0.31 to -0.05)	0.009	0.011	0.26 (504)
Age	-0.38 (-0.51 to -0.24)	1×10^{-7}	6×10^{-7}	
	**Slope HV ~ lateral orbitofrontal cMD + age + sex**			
Lateral orbitofrontal cMD	-0.19 (-0.33 to -0.05)	0.007	0.010	0.26 (503)
Age	-0.36 (-0.50 to -0.22)	1×10^{-6}	2×10^{-6}	
	**Slope HV ~ middle temporal cMD + age + sex**			
Middle temporal cMD	-0.22 (-0.38 to -0.07)	0.005	0.009	0.26 (503)
Age	-0.32 (-0.47 to -0.16)	9×10^{-5}	1×10^{-4}	
	**Slope HV ~ parahippocampal cMD + age + sex**			
Parahippocampal cMD	-0.18 (-0.31 to -0.04)	0.011	0.011	0.26 (504)
Age	-0.38 (-0.51 to -0.24)	2×10^{-7}	6×10^{-7}	

All models were adjusted for sex, which was a non-significant predictor. Multiple-comparisons corrected results are indicated by FDR *q* values.

Supplementary Table 7. Associations of regional cMD at baseline with longitudinal decline in hippocampal volume, accounting for entorhinal ^{18}F-flortaucipir.

Independent predictors	Std. β (95% CI)	*P* value	*q* value	R^2 (AIC)
	**Slope HV ~ entorhinal cMD + additional predictors**			
Entorhinal cMD	-0.09 (-0.21 to 0.03)	0.13	0.13	0.39 (471)
PIB-FLR	-0.21 (-0.33 to -0.08)	0.002	0.005	
Entorhinal CTh	0.17 (0.04 to 0.30)	0.010	0.082	
entFTP	-0.17 (-0.31 to -0.03)	0.019	0.082	
Age	-0.25 (-0.38 to -0.13)	1×10^{-4}	6×10^{-4}	
	**Slope HV ~ fusiform cMD + additional predictors**			
Fusiform cMD	-0.23 (-0.37 to -0.08)	0.003	0.020	0.39 (473)
PIB-FLR	-0.19 (-0.31 to -0.06)	0.004	0.005	
Fusiform CTh	-0.06 (-0.19 to 0.07)	0.38	0.51	
entFTP	-0.23 (-0.37 to -0.10)	0.001	0.001	
Age	-0.21 (-0.35 to -0.07)	0.004	0.006	
	**Slope HV ~ inf. parietal cMD + additional predictors**			
Inf. parietal cMD	-0.21 (-0.37 to -0.05)	0.009	0.020	0.38 (475)
PIB-FLR	-0.19 (-0.32 to -0.07)	0.003	0.005	
Inf. parietal CTh	-0.06 (-0.20 to 0.08)	0.38	0.51	
entFTP	-0.24 (-0.37 to -0.11)	5×10^{-4}	0.001	
Age	-0.21 (-0.36 to -0.06)	0.005	0.006	
	**Slope HV ~ inf. temporal cMD + additional predictors**			
Inf. temporal cMD	-0.23 (-0.39 to -0.06)	0.007	0.020	0.38 (475)
PIB-FLR	-0.19 (-0.32 to -0.06)	0.004	0.005	
Inf. temporal CTh	-0.08 (-0.21 to 0.06)	0.27	0.51	
entFTP	-0.23 (-0.36 to -0.09)	0.001	0.001	
Age	-0.19 (-0.34 to -0.04)	0.012	0.013	
	**Slope HV ~ isthmus cingulate cMD + additional predictors**			
Isthmus cingulate cMD	-0.15 (-0.28 to -0.03)	0.016	0.026	0.38 (475)
PIB-FLR	-0.20 (-0.32 to -0.07)	0.003	0.005	
Isthmus cingulate CTh	-0.07 (-0.19 to 0.05)	0.25	0.51	
entFTP	-0.25 (-0.38 to -0.11)	3×10^{-4}	0.001	
Age	-0.27 (-0.40 to -0.13)	2×10^{-4}	6×10^{-4}	
	**Slope HV ~ lat. orbitofrontal cMD + additional predictors**			
Lat. orbitofront. cMD	-0.15 (-0.29 to -0.02)	0.026	0.035	0.37 (477)
PIB-FLR	-0.20 (-0.33 to -0.08)	0.002	0.005	
Lat. orbitofront. CTh	-0.04 (-0.15 to 0.08)	0.52	0.59	
entFTP	-0.22 (-0.36 to -0.09)	0.001	0.002	
Age	-0.24 (-0.38 to -0.11)	6×10^{-4}	0.001	
	**Slope HV ~ middle temp. cMD + additional predictors**			
Middle temp. cMD	-0.21 (-0.38 to -0.05)	0.010	0.020	0.38 (476)
PIB-FLR	-0.19 (-0.32 to -0.06)	4×10^{-4}	0.005	
Middle temp. CTh	-0.10 (-0.24 to 0.04)	0.16	0.51	
entFTP	-0.24 (-0.38 to -0.11)	5×10^{-4}	0.001	
Age	-0.22 (-0.36 to -0.07)	0.005	0.006	
	**Slope HV ~ parahippocampal cMD + additional predictors**			
Parahipp. cMD	-0.11 (-0.25 to 0.03)	0.13	0.13	0.37 (479)
PIB-FLR	-0.18 (-0.31 to -0.05)	0.006	0.006	
Parahipp. CTh	0.03 (-0.10 to 0.15)	0.70	0.70	
entFTP	-0.24 (-0.37 to -0.10)	6×10^{-4}	0.001	
Age	-0.26 (-0.40 to -0.13)	2×10^{-4}	6×10^{-4}	

Multivariable regression models include as independent predictors: PIB-FLR, PACC5, regional CTh, entFTP and age; all models are adjusted by sex, which was a non-significant predictor. Multiple-comparisons corrected results are indicated by FDR *q* values.

Supplementary Table 8. Associations of regional cMD at baseline with longitudinal decline in hippocampal volume, accounting for inferior temporal ^{18}F-flortaucipir.

Independent predictors	Std. β (95% CI)	*P* value	*q* value	R^2 (AIC)
	**Slope HV ~ entorhinal cMD + additional predictors**			
Entorhinal cMD	-0.10 (-0.22 to 0.02)	0.12	0.13	0.38 (476)
PIB-FLR	-0.24 (-0.37 to -0.12)	2×10^{-4}	3×10^{-4}	
Entorhinal CTh	0.21 (0.08 to 0.33)	0.001	0.010	
i-tFTP	-0.08 (-0.21 to 0.06)	0.29	0.30	
Age	-0.26 (-0.39 to -0.13)	2×10^{-4}	5×10^{-4}	
	**Slope HV ~ fusiform cMD + additional predictors**			
Fusiform cMD	-0.23 (-0.38 to -0.07)	0.004	0.023	0.35 (484)
PIB-FLR	-0.24 (-0.37 to -0.12)	2×10^{-4}	3×10^{-4}	
Fusiform CTh	-0.03 (-0.17 to 0.10)	0.61	0.61	
i-tFTP	-0.08 (-0.23 to 0.07)	0.30	0.30	
Age	-0.24 (-0.38 to -0.09)	0.001	0.002	
	**Slope HV ~ inf. parietal cMD + additional predictors**			
Inf. parietal cMD	-0.21 (-0.37 to -0.05)	0.012	0.023	0.34 (486)
PIB-FLR	-0.25 (-0.37 to -0.12)	2×10^{-4}	3×10^{-4}	
Inf. parietal CTh	-0.07 (-0.21 to 0.08)	0.38	0.60	
i-tFTP	-0.12 (-0.26 to 0.03)	0.11	0.25	
Age	-0.24 (-0.39 to -0.09)	0.002	0.003	
	**Slope HV ~ inf. temporal cMD + additional predictors**			
Inf. temporal cMD	-0.24 (-0.41 to -0.07)	0.007	0.023	0.35 (485)
PIB-FLR	-0.24 (-0.37 to -0.12)	3×10^{-4}	3×10^{-4}	
Inf. temporal CTh	-0.08 (-0.22 to 0.06)	0.24	0.50	
i-tFTP	-0.10 (-0.24 to 0.05)	0.21	0.27	
Age	-0.22 (-0.37 to -0.06)	0.006	0.006	
	**Slope HV ~ isthmus cingulate cMD + additional predictors**			
Isthmus cingulate cMD	-0.14 (-0.27 to -0.01)	0.031	0.041	0.34 (486)
PIB-FLR	-0.25 (-0.38 to -0.12)	2×10^{-4}	3×10^{-4}	
Isthmus cingulate CTh	-0.08 (-0.20 to 0.05)	0.23	0.50	
i-tFTP	-0.12 (-0.26 to 0.02)	0.11	0.25	
Age	-0.30 (-0.44 to -0.15)	8×10^{-5}	4×10^{-4}	
	**Slope HV ~ lat. orbitofrontal cMD + additional predictors**			
Lat. orbitofront. cMD	-0.18 (-0.31 to -0.04)	0.013	0.023	0.35 (486)
PIB-FLR	-0.25 (-0.38 to -0.12)	1×10^{-4}	3×10^{-4}	
Lat. orbitofront. CTh	-0.04 (-0.16 to 0.08)	0.52	0.61	
i-tFTP	-0.11 (-0.25 to 0.03)	0.14	0.25	
Age	-0.26 (-0.40 to -0.11)	6×10^{-4}	0.001	
	**Slope HV ~ middle temp. cMD + additional predictors**			
Middle temp. cMD	-0.21 (-0.38 to -0.04)	0.014	0.023	0.34 (486)
PIB-FLR	-0.25 (-0.37 to -0.12)	2×10^{-4}	3×10^{-4}	
Middle temp. CTh	-0.08 (-0.23 to 0.06)	0.25	0.50	
i-tFTP	-0.11 (-0.26 to 0.04)	0.15	0.25	
Age	-0.24 (-0.39 to -0.09)	0.003	0.003	
	**Slope HV ~ parahippocampal cMD + additional predictors**			
Parahipp. cMD	-0.10 (-0.24 to 0.05)	0.18	0.18	0.34 (489)
PIB-FLR	-0.23 (-0.36 to -0.10)	5×10^{-4}	5×10^{-4}	
Parahipp. CTh	0.04 (-0.09 to 0.17)	0.54	0.61	
i-tFTP	-0.11 (-0.26 to 0.03)	0.12	0.25	
Age	-0.29 (-0.43 to -0.15)	1×10^{-4}	4×10^{-4}	

Linear regression models include as independent predictors: PIB-FLR, PACC5, regional CTh, inferior temporal FTP (i-tFTP) and age; all models are adjusted by sex, which was a non-significant predictor. Multiple-comparisons corrected results are indicated by FDR *q* values.

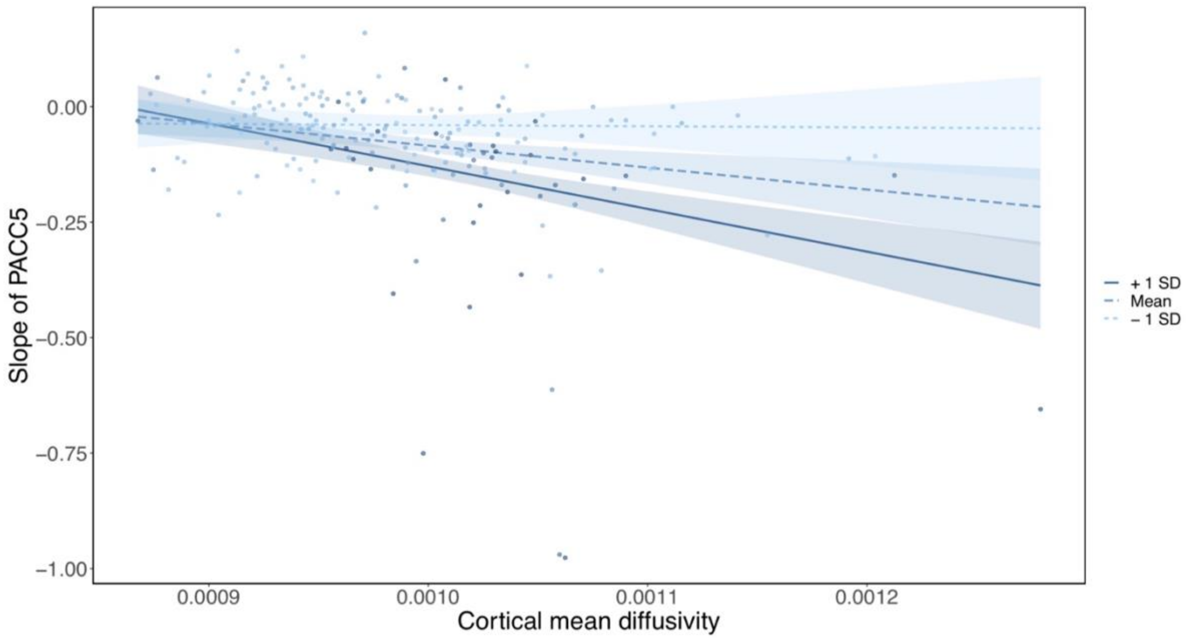

Supplementary Fig. 1. Interaction plot illustrating the association of cMD with the rate of cognitive decline at different levels of amyloid-β burden. The interaction plot illustrates the interaction effect at three levels of amyloid-β burden: mean PIB-FLR, mean PIB-FLR +1 SD and mean PIB-FLR - 1 SD.

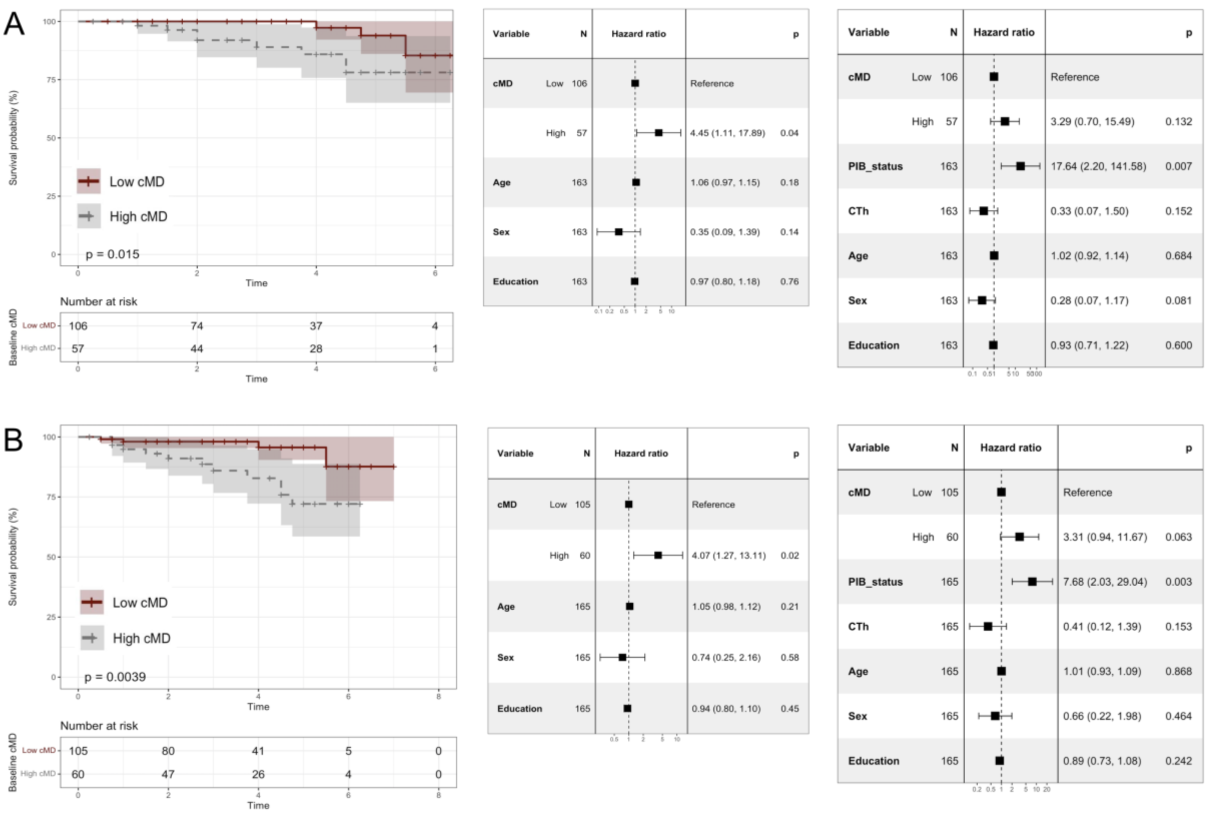

Supplementary Fig. 2. Survival analyses illustrating the ability of cMD in entorhinal cortex to predict subsequent clinical progression. Kaplan-Meier curves and Cox proportional hazards regression results for dichotomous entorhinal cMD (high/low cMD groups) predicting **(A)** progression to MCI**, (B)** progression to CDR = 0.5.

cMD = cortical mean diffusivity; CTh = cortical thickness; PIB_status = Aβ+ vs Aβ-; "High cMD" = top-tertile cMD values ("Low cMD", otherwise).

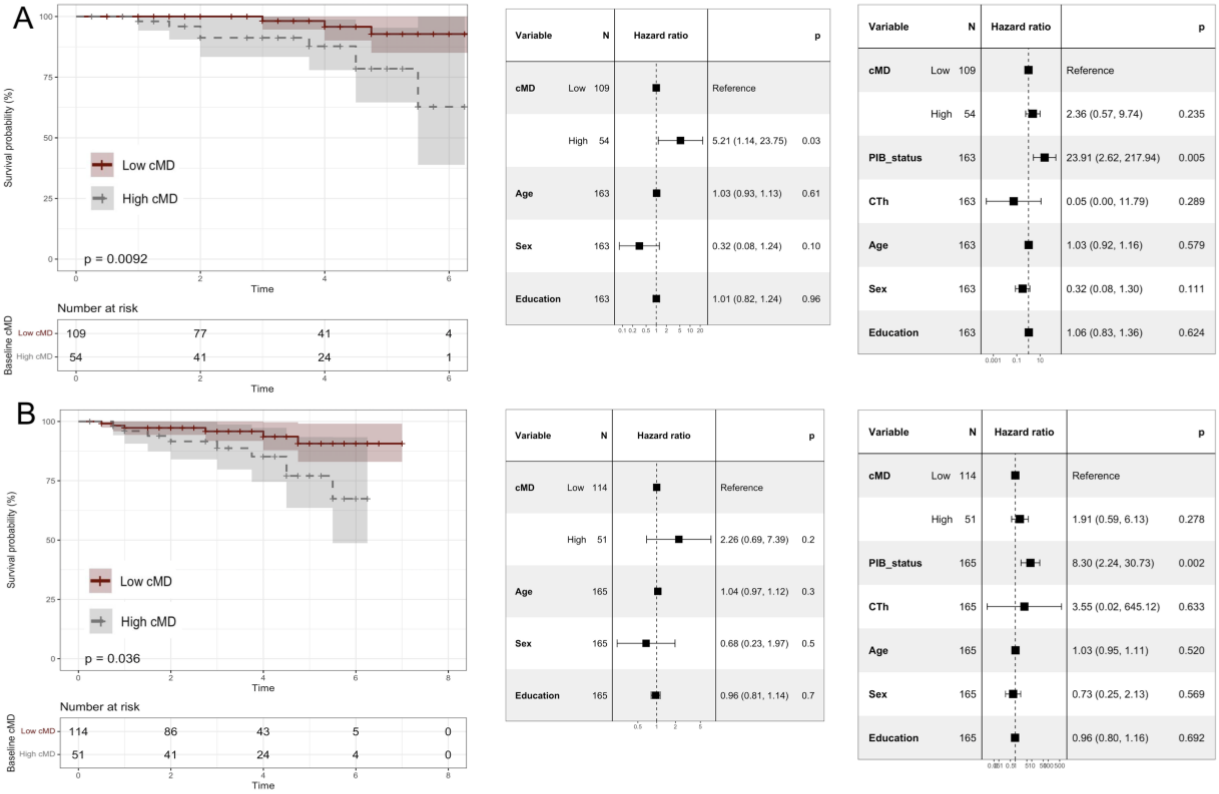

Supplementary Fig. 3. Survival analyses illustrating the ability of cMD in middle temporal cortex to predict subsequent clinical progression. Kaplan-Meier curves and Cox proportional hazards regression results for dichotomous middle temporal cMD (high/low cMD groups) predicting **(A)** progression to MCI**, (B)** progression to CDR = 0.5.

cMD = cortical mean diffusivity; CTh = cortical thickness; PIB_status = Aβ+ vs Aβ-; "High cMD" = top-tertile cMD values ("Low cMD", otherwise)
